# Pseudohyphal differentiation in *Komagataella phaffii*: investigating the *FLO* gene family

**DOI:** 10.1093/femsyr/foaa044

**Published:** 2020-08-07

**Authors:** Sonakshi De, Corinna Rebnegger, Josef Moser, Nadine Tatto, Alexandra B Graf, Diethard Mattanovich, Brigitte Gasser

**Affiliations:** Austrian Centre of Industrial Biotechnology, Muthgasse 11, 1190 Vienna, Austria; Department of Biotechnology, BOKU University of Natural Resources and Life Sciences, Muthgasse 18, 1190 Vienna, Austria; Department of Biotechnology, BOKU University of Natural Resources and Life Sciences, Muthgasse 18, 1190 Vienna, Austria; CD-Laboratory for Growth-decoupled Protein Production in Yeast, BOKU University of Natural Resources and Life Sciences, Muthgasse 18, 1190 Vienna, Austria; Austrian Centre of Industrial Biotechnology, Muthgasse 11, 1190 Vienna, Austria; School of Bioengineering, University of Applied Sciences-FH Campus Wien, Muthgasse 11, 1190 Vienna, Austria; Austrian Centre of Industrial Biotechnology, Muthgasse 11, 1190 Vienna, Austria; Department of Biotechnology, BOKU University of Natural Resources and Life Sciences, Muthgasse 18, 1190 Vienna, Austria; Austrian Centre of Industrial Biotechnology, Muthgasse 11, 1190 Vienna, Austria; School of Bioengineering, University of Applied Sciences-FH Campus Wien, Muthgasse 11, 1190 Vienna, Austria; Austrian Centre of Industrial Biotechnology, Muthgasse 11, 1190 Vienna, Austria; Department of Biotechnology, BOKU University of Natural Resources and Life Sciences, Muthgasse 18, 1190 Vienna, Austria; Austrian Centre of Industrial Biotechnology, Muthgasse 11, 1190 Vienna, Austria; Department of Biotechnology, BOKU University of Natural Resources and Life Sciences, Muthgasse 18, 1190 Vienna, Austria; CD-Laboratory for Growth-decoupled Protein Production in Yeast, BOKU University of Natural Resources and Life Sciences, Muthgasse 18, 1190 Vienna, Austria

**Keywords:** *Komagataella phaffii*, pseudohyphal growth, specific growth rate, FAIRE-Seq, epigenetics, *FLO* genes, *Pichia pastoris*

## Abstract

Many yeasts differentiate into multicellular phenotypes in adverse environmental conditions. Here, we investigate pseudohyphal growth in *Komagataella phaffii* and the involvement of the flocculin (*FLO*) gene family in its regulation. The *K. phaffii FLO* family consists of 13 members, and the conditions inducing pseudohyphal growth are different from *Saccharomyces cerevisiae*. So far, this phenotype was only observed when *K. phaffii* was cultivated at slow growth rates in glucose-limited chemostats, but not upon nitrogen starvation or the presence of fusel alcohols. Transcriptional analysis identified that *FLO11*, *FLO400* and *FLO5-1* are involved in the phenotype, all being controlled by the transcriptional regulator Flo8. The three genes exhibit a complex mechanism of expression and repression during transition from yeast to pseudohyphal form. Unlike in *S. cerevisiae*, deletion of *FLO11* does not completely prevent the phenotype. In contrast, deletion of *FLO400* or *FLO5-1* prevents pseudohyphae formation, and hampers *FLO11* expression. FAIRE-Seq data shows that the expression and repression of *FLO400* and *FLO5-1* are correlated to open or closed chromatin regions upstream of these genes, respectively. Our findings indicate that *K. phaffii* Flo400 and/or Flo5-1 act as upstream signals that lead to the induction of *FLO11* upon glucose limitation in chemostats at slow growth and chromatin modulation is involved in the regulation of their expression.

## Teaser

Two novel members of the *FLO* gene family, *FLO400* and *FLO5-1*, are involved in pseudohyphal growth in *Komagataella phaffii*.

## INTRODUCTION

Yeasts possess the ability to exhibit morphological and physiological differentiations upon experiencing adverse environmental conditions. Such differentiations can give rise to multiple subpopulations of cells exhibiting different phenotypes from a homogeneous population, thus increasing chances of survival either of individual cells or an entire subpopulation. Depending on the environmental cue, such differentiations may include switching from budded yeast form to filamentous form, flocculation, sporulation and programmed cell death (Schneper *et al*. 2004; Zaman *et al*. 2008). Cell differentiation is driven by interconnected complex signaling networks regulated by a plethora of transcription factors and chromatin regulators like histone-modifying enzymes and chromatin remodelers (Jaiswal *et al*. 2017). Both pathogenic and non-pathogenic yeasts feature such cell differentiation. For example, in the fungal pathogen *Candida albicans*, signals such as neutral pH, body temperature, serum, nutrient availability, etc. can trigger a switch from yeast to filamentous form, which is necessary for its virulence (Biswas *et al*. 2007). Also, baker's yeast *Saccharomyces cerevisiae* is known to exhibit differentiation in response to nutrient limitation and other environmental triggers. Such phenotypic differentiation is often driven by families of genes encoding cell-surface proteins, such as the *ALS* gene family in *C. albicans* or the *FLO* gene family in *S. cerevisiae*.

The *FLO* gene family of *S. cerevisiae* is known to have five dominant members: *FLO1*, *FLO5*, *FLO9*, *FLO10* and *FLO11* (Teunissen and Steensma 1995; Caro *et al*. 1997). Out of these, *FLO1*, *FLO5*, *FLO9* and *FLO10* share sequence homology, are located adjacent to telomeres and are known to promote cell–cell adhesion, commonly called flocculation (Guo *et al*. 2000). *FLO11* on the other hand is known to be responsible for filamentous growth whereby cells divide but remain adhered to each other, thus forming a long chain of filament-like structures (Lambrechts *et al*. 1996; Lo and Dranginis 1998). Filamentous growth can either result in filaments spreading on the surface, a phenomenon termed as pseudohyphal growth, or downward extension of filaments into the solid substrate, known as invasive growth. While both haploid and diploid cells can exhibit filamentous growth, pseudohyphal growth is more prevalent in diploid cells and invasive growth is more prevalent in haploid cells of *S. cerevisiae* (Wright *et al*. 1993; Cullen and Sprague 2000; Song and Kumar 2012).

In *S. cerevisiae*, flocculation is triggered by external stressors like antimicrobials and other chemical agents, temperature or pH variations (Smukalla *et al*. 2008). Pseudohyphae formation or invasive growth is triggered by nitrogen and/or glucose starvation or exposure to fusel alcohols (Gimeno *et al*. 1992; Dickinson 1996). Depending on the genetic background and external trigger, the expression of one or more of the *FLO* family members may be affected. The commonly used laboratory strain S288C is impaired in pseudohyphal growth, flocculation, invasive growth and biofilm formation because it carries a nonsense mutation in Flo8p, which is a key transcriptional activator of the *FLO* genes (Liu *et al*. 1996). Most of the *S. cerevisiae* studies related to adhesion have been carried out in the ∑1278b strain that has an intact functional Flo8p and in which only *FLO11* is active, while the other *FLO* genes are telomerically silenced (Halme *et al*. 2004).

Not much was known about flocculation and pseudohyphae forming behavior in the methylotrophic yeast and popular protein production host *Komagataella phaffii* (syn. *Pichia pastoris*) until Rebnegger *et al*. reported that *K. phaffii* switches to pseudohyphal growth and exhibits surface adherence when cultivated at slow growth rates in glucose-limited chemostats (Rebnegger *et al*. 2014, 2016). The authors observed that at growth rates of *µ* = 0.075 h^−1^ or below, some of the cells changed their morphological appearance adopting an elongated shape or occasionally a branched pseudohyphae. As the elongated phenotype persisted after switching back to faster growth rates, they speculated that the transition of *K. phaffii* from yeast to pseudohyphal form might be epigenetically regulated (Rebnegger *et al*. 2014).

*Komagataella phaffii* also has a *FLO* gene family but its members and their functions are yet to be explored. The proteins encoded by these genes show very low sequence similarity to each other or to the *S. cerevisiae FLO* proteins making it difficult to predict which of them participate in imparting pseudohyphal growth or other adhesion-related phenotypes. As seen from studies in *S. cerevisiae* (Govender *et al*. 2008; Moreno-García *et al*. 2018; Westman *et al*. 2018), knowledge about these genes can open up paths for engineering strains that are more suited for industrial production processes. Prompted by the observations reported in the abovementioned studies by Rebnegger *et al*., we decided to take a closer look at the *FLO* gene family in *K. phaffii* in order to identify the gene or genes responsible for conferring this pseudohyphal phenotype.

## MATERIALS AND METHODS

### Strains and strain creation

All *K. phaffii* strains used in this study (Table 1) are based on the CBS7435 wild-type strain. The CBS7435 *flo8Δ* used here was the same strain described in Rebnegger *et al*. (Rebnegger *et al*. 2016). All the deletion strains were created using the split-marker cassette method adapted for *K. phaffii* (Fairhead *et al*. 1996; Gasser *et al*. 2013). Using this method, the target genes were replaced by an expression cassette encoding an antibiotic selection marker flanked by around 1000 bp of homologous regions for integration. The homologous flanking regions for creating the deletion cassette for *flo11Δ* and *flo5-1Δ* were amplified from the *K. phaffii* genomic DNA using primers containing *Bsa*I restriction sites and also fusion sites (FS) for Golden Gate Cloning (FS A–B for 5′ region and FS C–D for 3′ region) (Prielhofer *et al*. 2017). The marker sequences were amplified from available plasmids using primers for introduction of *Bsa*I and FS B-C. In the case of *Δflo400*, the homologous region contained multiple *Bsa*I restriction sites. Therefore, the primers used for the amplification of these regions were designed to contain *Bpi*I restriction sites and fusion sites FS 1–2 and FS 3–4 for 5′ and 3′ regions, respectively. Accordingly, the marker sequence was amplified with primers to introduce *Bpi*I restriction sites and FS 2–3. All marker cassettes were flanked by *loxP* sites for marker recycling using Cre recombinase. Golden Gate cloning was then carried out using *Bsa*I or *Bpi*I to assemble a vector containing the entire deletion cassette. The vector was used as a template for PCR to generate two fragments overlapping in the marker gene sequence. Finally, 500 ng of these two fragments were transformed into electrocompetent *K. phaffii* to construct a single deletion or CBS7435 *Δflo11* cells to construct double deletions using the transformation protocol described previously (Gasser *et al*. 2013). Correct deletions were identified by colony PCR using forward and backward primers located outside the flanking homologous regions. For the *flo8Δ* and *flo11Δ* strains, the marker cassette was removed by transforming the cells with 300 ng of circular pKTAC_Cre_hph encoding the Cre recombinase as described by Marx, *et al*. (Marx *et al*. 2008). All enzymes used in this study were purchased from New England Biolabs (Frankfurt, Germany) except for *Bpi*I which was purchased from Thermo Fisher Scientific (Vienna, Austria).

**Table 1.** Strains used in this study.

Strain	Genotype	Resistance	
CBS7435	Wild type		
CBS7435 *flo8Δ*	*flo8Δ* ^a^	None	Rebnegger *et al*. (2016)
CBS7435 *flo11Δ*	*flo11Δ::loxP-natMX-loxP*	Nourseothricin	This study
CBS7435 *flo400Δ*	*flo400Δ::loxP-kanMX-loxP*	Geneticin	This study
CBS7435 *flo5-1Δ*	*flo5-1Δ::loxP-kanMX-loxP*	Geneticin	This study
CBS7435 *flo11Δ flo400Δ*	*flo11Δ flo400Δ::loxP-kanMX-loxP* ^b^	Geneticin	This study
CBS7435 *flo11Δ flo5-1Δ*	*flo11Δ flo5-1Δ::loxP-kanMX-loxP* ^b^	Geneticin	This study
CBS7435 *FLO11-eGFP*	*pFLO11-5*′*FLO11(69bp)-linker-eGFP-linker-FLO11*^c^	None	This study
CBS7435 *FLO400-eGFP*	*pFLO400-5*′*FLO400(54bp)-linker-eGFP-linker-FLO400*^c^	None	This study
CBS7435 *FLO5-1-eGFP*	*pFLO5-1-5*′*FLO5-1(78bp)-linker-eGFP-linker-FLO5-1*^c^	None	This study

a *flo8Δ* strain has a disrupted PP7435_Chr4-0252 locus with 221 bp of the promoter directly upstream of the ORF and the first 228 bp (76 amino acids) deleted.

b *flo11Δ flo400Δ* and *flo11Δ flo5-1Δ* strains have a *loxP-kanMX-loxP* cassette in the *FLO400*and*FLO5-1* locus, respectively, and an additional *loxP* sequence in the *FLO11* locus.

c These strains were constructed using CRISPR-Cas9 technique to add the enhanced green fluorescent protein (eGFP) sequence in the native locus after the cleavage site of the pre-sequence.

To create the *FLO11-eGFP*, *FLO400-eGFP* and *FLO5-1-eGFP* strains, CRISPR/Cas9 mediated homology-directed repair was employed (Gassler *et al*. 2019). Since all the three proteins contain a signal peptide pre-sequence and a signal peptidase cleavage site, the enhanced green fluorescent protein (eGFP) sequence was added internally after the pre-sequence at the amino-terminal as described for *S. cerevisiae* Flo11 (Lo and Dranginis 1998). To generate the 5′ homologous region, the region around 1000 bp upstream including the start codon and the pre-sequence was selected and amplified by PCR using genomic DNA as template. Similarly, for the 3′ homologous region 1000 bp of the gene sequence after the pre-sequence was amplified. A vector containing the eGFP sequence was used as a template to amplify the eGFP fragment. The primers used for this purpose were designed to add a AAA linker before and after the eGFP sequence. As described above, all the fragments contained *Bsa*I sites in case of *FLO5-1* or *Bpi*I sites in case of *FLO400*. Additionally, the 5′ end and the 3′ end of the donor DNA also contained an additional restriction site (*Sap*I for *FLO5-1-eGFP*, and *Bsm*I for *FLO400-eGFP* constructs) to enable excision of the donor DNA from the vector. Golden Gate assembly was used to assemble a BB3 vector containing the eGFP coding sequence flanked by the 5′ and 3′ homologous regions. The assembled vector was transformed into chemically competent *Escherichiacoli* cells. Sufficient amount of plasmid was extracted from the obtained *E. coli* clones and sequencing was carried out to confirm that the donor sequence is correct. The donor was then excised out of the vector. Human codon-optimized Cas9 under the control of *K. phaffii LAT1* promoter and a guide RNA targeting the region directly upstream of the start codon under the control of the pGAP were expressed from an episomal plasmid vector (Gassler *et al*. 2019) and 500 ng of this vector was transformed along with the donor DNA for integration of the eGFP sequence. Correct integration was checked by PCR using primers binding outside the homologous regions and sequencing of the locus.

All primers used to generate the strains are listed in Table S6 (Supporting Information).

### Cultivations and sampling

All media components used in this work were purchased from Carl Roth (Karlsruhe, Germany) and Merck (Darmstadt, Germany) unless specified otherwise. Yeast cells were grown in standard yeast peptone (YP) medium (10 gL^−1^ yeast extract, 20 gL^−1^ soy peptone) containing 2% glucose as carbon source (YPD) and antibiotics in case of strains containing a selection marker. All shake flask cultures were grown at 25°C and 180 rpm. Cultures for cryostocks were grown overnight in 100 mL flasks containing 10 mL YPD with or without antibiotics. 10% vol/vol glycerol was added to 1 mL of overnight culture and stored at −80°C. Precultures for inoculation of chemostats were grown by thawing one cryostock of the required strain and adding to 1000 mL flasks containing 100 mL YPD with or without antibiotics.

For the nitrogen limitation experiments, minimal medium agar plates were prepared containing (L^−1^) 0.25 g MgSO_4_*7H_2_O, 0.40 g KCl, 0.0134 g CaCl_2_*2H_2_O, 11 g citric acid monohydrate, 735 µL trace element solution, 1 mL biotin stock solution (0.1 gL^−1^), supplemented with 2% glucose as carbon source. The trace salt solution contained (L^−1^) 5.0 g FeSO_4_·7H_2_O, 20.0 g ZnCl_2_, 6.0 g CuSO_4_·5H_2_O, 3.36 g MnSO_4_·H_2_O, 0.82 g CoCl_2_*6H_2_O, 0.2 g Na_2_MoO_4_·2H_2_O, 0.08 g NaI, 0.02 g H_3_BO_3_ and 5.0 mL H_2_SO_4_ (95–98% w/w). The amount of (NH_4_)_2_HPO_4_ was adjusted depending on the target concentration of nitrogen (1.58 g for standard conditions of 24 mM and 0.0033 g for final nitrogen concentration of 50 µM). The pH was adjusted to 5.5 with KOH. The medium was sterile filtered and 250 mL of 4% agar was added under sterile conditions prior to use. For plating of the cells, precultures were diluted to an OD_600_ units of 0.1, washed with sterile water to remove residual nutrients and spread on the required plate (YP agar for glucose limitation and low nitrogen agar for nitrogen limitation). As control, cells were simultaneously also plated on YPD agar plates and standard minimal agar plates. The plates were incubated at 25°C for 3 days and observed for morphological changes by microscopy.

For cultivation in the presence of fusel alcohols, 10 mL of YPD medium was inoculated with cells to a final OD_600_ units of 0.1 and either 100 µL of butanol (final concentration of 1%) or 150 µL of isoamyl alcohol (final concentration of 1.5%) was added. The flasks were incubated at 25°C for 3 days with shaking at 180 rpm and the cultures were checked daily for morphological changes by microscopy.

Glucose-limited chemostat cultivation was carried out as described in Rebnegger *et al*. (Rebnegger *et al*. 2016). Two different dilution rates (0.1 h^−1^ and 0.05 h^−1^) were used and the cultivations were carried out in 1 L DASGIP benchtop bioreactors (SR0700ODLS; Eppendorf, Hamburg, Germany). Precultures were grown overnight as described in the previous section. Cells were then harvested, washed, resuspended in sterile demineralized water and finally used to inoculate bioreactors at a final OD_600_ units of 2. The chemostat medium contained (L^−1^) 2 g citric acid monohydrate, 44 g glucose, 17.4 g (NH4)_2_HPO_4_, 0.8 g MgSO_4_*7H_2_O, 2 g KCl, 0.03 g CaCl_2_*2H_2_O, 1.94 g trace element solution, 0.5 g Pluronic and 3.2 g biotin stock solution (0.1 gL^−1^). The pH was set to 5.85 with 20% HCl. The trace salt solution used was the same as described before.

After completion of the batch phase, which was indicated by a sharp rise in dissolved oxygen concentration, the chemostat was initiated at a dilution rate of 0.1 h^−1^, then switched to 0.05 h^−1^ and finally switched back to 0.1 h^−1^. The chemostat was run for five resident times at each dilution rate set point to attain steady state conditions. The working volume was kept steady at 400 mL by means of a level sensor. Cultures were stirred at 700 rpm and supplied with a constant airflow of 25 SLh^−1^ to keep the dissolved oxygen concentration above 20%. The culture temperature was set to 25°C and the pH was kept at 5.85 by the addition of 12.5% ammonia solution. At every sampling time point (Fig. 3A), samples were collected for the required analyses such as OD_600_ measurement, FAIRE-Seq, transcriptomics (qRT-PCR and RNA-Seq) and microscopy. Since the *flo8Δ* strain did not show any phenotype at the slow growth rate, RNA-Seq samples for this strain were taken only initially at the fast growth rate and then after switching to the slow growth rate. No samples for RNA-Seq were taken for this strain after switching back to the fast growth rate.

For formaldehyde fixation of samples for FAIRE-Seq, first the OD_600_ of the culture was measured. Then, fixation solution was prepared in a 500 mL shake flask that contained 100 mL PBS with 1% formaldehyde per 50 OD_600_ units of cells to be fixed. The required amount of bioreactor culture was drawn and directly added to the fixation solution. The flask was shaken at room temperature for ∼30 min, after which the formaldehyde was quenched by addition of 500 mM Tris–Cl (pH 8.0) and shaking for 5 min. Finally, cells were harvested, washed with PBS and stored at −80°C.

For fixing of cells for transcriptomics studies, samples were added in a 2:1 ratio to precooled fixing solution containing 5% (vol/vol) phenol in absolute ethanol. Samples were then centrifuged for 1 min at 10 000 × *g* and 4°C, afterward the supernatant was removed and the pellet was stored at −80°C until further processing.

### FAIRE-Seq

The FAIRE-Seq protocol was adapted from protocols published in the study by Nagy *et al* and Simon *et al* (Nagy *et al*. 2003; Simon *et al*. 2012). The formaldehyde-fixed cells were thawed and resuspended in 1 mL lysis buffer and transferred to 2 mL screwcap tubes containing 500 µL of acid-washed 500 µm glass beads. Cells were lysed in a FastPrep®-24 equipment (MP Biomedicals, CA, USA) for 5 × 40 s cycles, with 2 min rest in between. The lysate was transferred to a 15 mL conical tube. The glass beads were washed with an additional 600 µL lysis buffer, which was then added to the same conical tube. The lysate was sonicated in a Bioruptor® Plus (Diagenode, Liège, Belgium ) for 10 cycles (30 s on/30 s off) at high power setting. The sonicated lysate was clarified by centrifugation at 10 000 × *g* for 5 min and around 150 µL of this clarified lysate was removed for preparation of input control DNA. The remaining lysate was aliquoted into fresh 1.5 mL tubes. 1 volume of phenol/chloroform/isoamyl alcohol (Thermo Fisher Scientific, Vienna, Austria) was added to them, vortexed vigorously and centrifuged at full speed for 10 min and the top layer was transferred to a fresh tube. This was repeated one more time after which 200 µL of chloroform/isoamyl alcohol (Thermo Fisher Scientific, Vienna, Austria) was added to each tube to remove any remnant phenol. The tubes were vortexed, centrifuged and the aqueous layer transferred to a fresh tube. 1/10 volume 3M sodium acetate (pH 5.2), 2 volumes 95% ethanol and 1 µL 20 mg/mL glycogen was added to each tube and the tubes were incubated at −80°C overnight. The tubes were centrifuged at full speed for 20 min to precipitate the DNA, after which the pellets were washed with 75% ice-cold ethanol and centrifuged again for another 10 min. Finally, the ethanol was removed and the pellets dried and resuspended in 50 µL 10 mM Tris–HCl (pH 7.4). These FAIRE samples were treated with DNase-free RNase A (30 min, 37°C), Proteinase K (1 h, 55°C) and finally incubated overnight at 65°C for decrosslinking.

For preparation of input control DNA, the 150 µL clarified lysate removed after sonication was first treated with DNase-free RNase A, Proteinase K and decrosslinked overnight at 65°C before proceeding with Phenol/Chloroform/Isoamyl alcohol extraction as described for the FAIRE samples. Both the FAIRE DNA and input control DNA were further purified using MinElute PCR purification kit (Qiagen, Hilden, Germany), quantified and run on an agarose gel to check proper sonication efficiency.

Library preparation and DNA sequencing (Illumina HiSeq 2000, paired‐end, 50 bp read length) was performed at the VBCF NGS unit (www.vbcf.ac.at). Raw reads were filtered of adapter sequence and low-quality reads. Mapping over the *K. phaffii* CBS7435 genome was carried out with the BWA tool using default settings (Langmead and Salzberg 2012). Reads with mapping quality <20 were filtered out. The mapped data was uploaded on the public server usegalaxy.org (Afgan *et al*. 2018) and peak calling was carried out using MACS2 tool (Zhang *et al*. 2008) with setting the *P*-value cut-off as 1e-10. The replicate data was pooled and input control was used for peak calling. Comparison between the wild-type samples at different conditions and between the wild-type and the knock-out strains was done by using the ‘Join’ tool from ‘Operate on Genomic Intervals’ in Galaxy. To determine the genomic location of the peaks, the feature list for the *K. phaffii* CBS7435 strain was downloaded from http://www.pichiagenome.org (Valli *et al*. 2016) and the same join tool was used to identify which genes are located upstream or downstream closest to the peaks. Peaks were visualized with Integrated Genome Browser (IGB) software (Nicol *et al*. 2009).

### RNA extraction

The ethanol/phenol fixed cells were resuspended in 1 mL TRI reagent (Sigma-Aldrich, Darmstadt, Germany), lysed using glass beads as described in the previous section and RNA was extracted according to the TRI reagent protocol. DNase treatment was carried out using the Ambion DNA-free kit (Thermo Fisher Scientific, Vienna, Austria). RNA concentration and integrity were analyzed using the Nanodrop spectrophotometer and Bioanalyzer (Agilent, Vienna, Austria). cDNA was synthesized using Oligo(dt)_23_ primers (NEB, Frankfurt, Germany) and Biozym cDNA synthesis kit (Biozym, Vienna, Austria). Quantitative PCR was carried out using Biozym Blue Probe qPCR kit on a Rotor Gene Q instrument (Qiagen, Hilden, Germany). Changes in transcript levels were calculated relative to the reference sample, after normalization to *ACT1* (PP7435_Chr3-0993) expression, using the threshold cycle method of the Rotor Gene software.

### RNA sequencing

The poly-A, single read RNA Sequencing of the five samples, each with three replicates, was conducted at the Vienna Bioinformatics Core Facility NGS Unit (www.vbcf.ac.at) on an Illumina HiSeq V4. Sorting of the received unsorted and unaligned BAM files was performed by using samtools sort v1.5 (Li *et al*. 2009). Subsequently the conversion into FASTQ files was executed with picard tools v2.17.3 SamToFastq (Broad Institute 2018). Afterward the reads were quality checked and trimmed by utilizing TrimGalore v0.4.2 (Krueger 2012), which itself relies on FastQC v0.11.5 (Andrews and Fast 2010) and Cutadapt (Martin 2010). As the count quantification with kallisto depends on a transcript index of the reference sequences, this index was created with kallisto index v0.43.1 (Bray *et al*. 2016) and the latest *K. phaffii* CBS7435 annotation (FR839628.1, FR839629.1, FR839630.1, FR839631.1, FR839632.1). Accordingly, the count data of all sample reads was calculated with kallisto quant v0.43.1. At the same time pseudobam files were created within the same run for each sample using the gtf file of *K. phaffii* CBS 7435 (*Komagataella phaffii*_cbs_7435.PicPas_Mar2011.38.gtf.gz, October 2016) from Ensembl Fungi (Kersey *et al*. 2018) as reference.

Differential expression analysis (Love *et al*. 2014) for each sample comparison was performed with several R v3.3.2 packages, which are tximport and tximportData v1.2.0 (Soneson *et al*. 2015), readr v1.1.1 (Wickham, Hester and Francois 2015) and DESeq2 v1.14.1 (Gentleman *et al*. 2004; Love *et al*. 2014; Huber *et al*. 2015). Transcripts with a log2 fold-change below −1 and above 1, combined with an adjusted *P*-value below 0.05 were considered to be significantly expressed.

### Microscopy

Microscopy was performed with a Zeiss Axio Observer microscope using a LCI Plan-Neofluar 63X (numerical aperture 1.3) water immersion objective in differential interference contrast (DIC) mode and using the 38 HE eGFP shift free (E) filter set (Carl Zeiss, Oberkochen, Germany). Images were processed using ImageJ 1.52i software (Schneider *et al*. 2012)

## RESULTS

### The *FLO* gene family of *K. phaffii*

**Figure 1.**
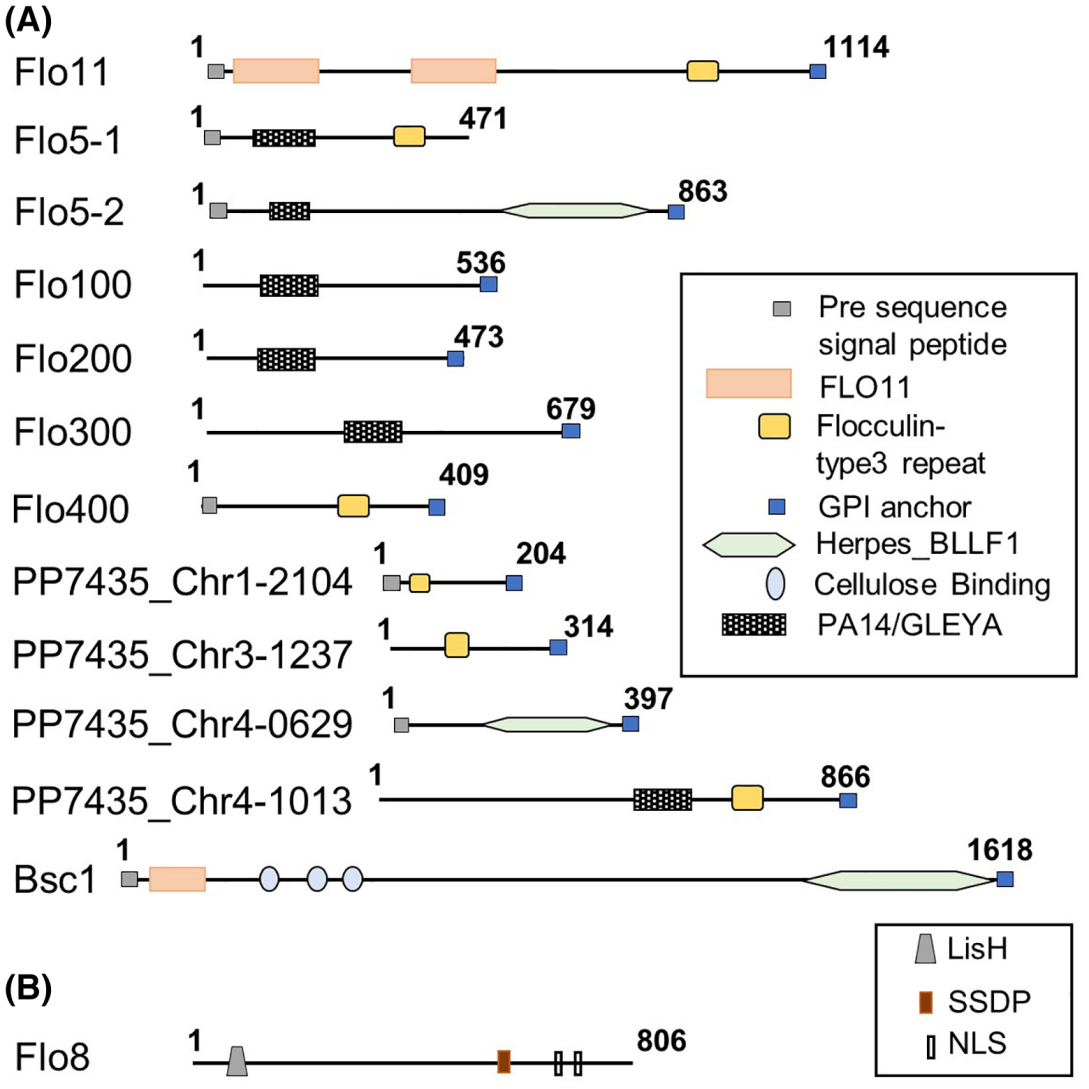
Schematic representation of the structures of the *K. phaffii FLO* family proteins. **(A)**Line diagrams representing the Flo proteins with their predicted N-terminal signal peptides, identified conserved domains and GPI anchor. **(B)** Representation of Flo8, master transcription regulator controlling several *FLO* genes harboring an N-terminal Lis homology (LisH) domain, a putative single‐stranded DNA‐binding protein (SSDP) domain and a nuclear localization signal (NLS).

In *K. phaffii* CBS7435 (Sturmberger *et al*. 2016; Valli *et al*. 2016), there are seven genes annotated as *FLO* genes, namely *FLO11* (PP7435_Chr2-0267)*, FLO5-1* (PP7435_Chr3-1389), *FLO5-2* (PP7435_Chr1-1228), *FLO100* (PP7435_Chr1-1587), *FLO200* (PP7435_Chr3-1226), *FLO300* (PP7435_Chr4-1020) and *FLO400* (PP7435_Chr4-0865). Their annotation was based on the fact that the proteins they encode carry conserved domains typical to flocculin proteins such as flocculin_t3, PA14/GLEYA and FLO11 (Fig. 1A; Table S1, Supporting Information). Thereof, Flo11 is the putative homolog of *S. cerevisiae* Flo11, while the other proteins have only low sequence similarity to the *S. cerevisiae* FLO proteins. Based on reciprocal BLASTp, two of the proteins had significant sequence similarity with Flo5, and are thus named Flo5-1 and Flo5-2, while for the others no clear *S. cerevisiae* homolog could be designated and the names Flo100-400 were chosen. Additionally, there are six other genes- *BSC1* (PP7435_Chr1-1549), PP7435_Chr1-2104, PP7435_Chr2-0004, PP7435_Chr3-1237, PP7435_Chr4-0629 and PP7435_Chr4-1013—that encode for proteins that share low sequence similarity with flocculin proteins (Valli *et al*. 2016). Among these genes, even though the protein product of PP7435_Chr2-0004 shares a low similarity with Flo5-1 and Flo5-2, no conserved domains, tandem repeats or glycosylphosphatidylinositol (GPI) anchors were identified in the protein and hence it was excluded from Fig. 1A.

Flo11 and Bsc1 carry N-terminal *FLO11* flocculin domains (Fig. 1A) like their *S. cerevisiae* homologs while the majority of the other Flo proteins carry a PA14/GLEYA domain, which is a carbohydrate binding domain found in fungal adhesins, containing a conserved motif G(M/L)(E/A/N/Q) (Linder and Gustafsson 2008). In Flo5-1, Flo5-2, Flo100 and Flo200 the domain is located closer to the N-terminus, while in Flo300 and in the protein encoded by PP7435_Chr4-1013 it is located toward the center or closer to the C-terminus, respectively. Here, among the PA14 domain containing Flo proteins, Flo5-1 and Flo5-2 have a predicted N-terminal signal peptide, while the others do not. Additionally, all of these proteins except for Flo5-1 carry a GPI anchor. Almost all the other remaining Flo proteins, namely Flo11, Flo400, Bsc1 and the protein products of  PP7435_Chr1-2104 and PP7435_Chr4-0629 carry an N-terminal signal peptide and a C-terminal GPI anchor. Here, an exception is PP7435_Chr3-1237, which has a predicted GPI anchor but no N-terminal signal peptide. Additionally, some of the proteins encoded by these *FLO* genes, namely Flo11, Flo5-1, Flo400 and the protein products of PP7435_Chr1-2104, PP7435_Chr3-1237 and PP7435_Chr4-1013 harbor a flocculin_t3 repeat, which is a repeat found in *S. cerevisiae* Flo9 close to its C-terminus (Marchler-Bauer *et al*. 2011). Some of the other Flo proteins, namely, Flo5-2, Bsc1 and the protein product of PP7435_Chr4-0629 carry a Herpes_BLLF1 domain close to their C-termini. As characteristic of flocculin proteins, most of the *K. phaffii* Flo proteins have a central domain containing multiple tandem repeats that are primarily rich in threonine and proline.

**Figure 2.**
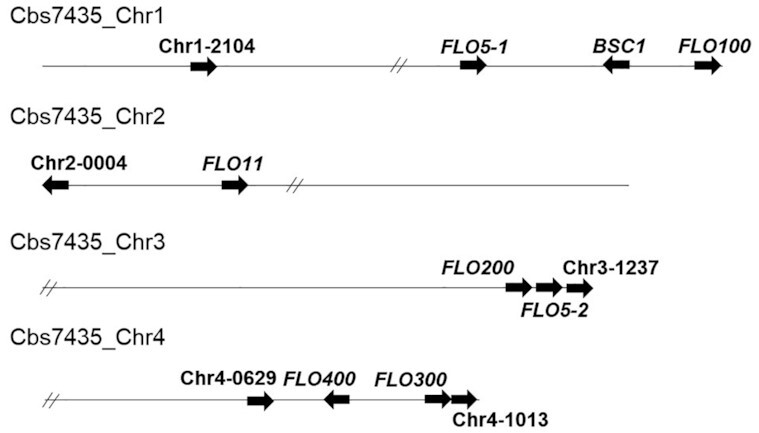
Location of the *FLO* genes on the chromosomes of *K. phaffii* CBS7435. // indicates the localization of the centromeres.

Analysis of the amino acid sequences show that Flo5-1 and Flo5-2 share >50% sequence similarity. Apart from this, the Flo proteins share very little sequence similarity to each other or to any of the *S. cerevisiae FLO* gene products. Like in *S. cerevisiae*, many of the *K. phaffii FLO* genes are located adjacent to telomeres, except for *FLO11*, *FLO5-1*, *FLO400*, PP7435_Chr1-2104 and PP7435_Chr4-0629 (Fig. 2). In *S. cerevisiae*, telomeric silencing of *FLO* genes has been described during standard growth conditions (Halme *et al*. 2004). Therefore, we analyzed the expression levels of all *FLO* genes in RNA sequencing data of *K. phaffii* cultivated in shake-flasks in minimal medium containing 5 g L^−1^ glucose that was previously carried out in our lab (Ata *et al*. 2018). In this condition, some of the *FLO* genes, namely *FLO5-1*, *FLO5-2*, *FLO300*, PP7435_Chr1-2104, PP7435_Chr3-1237, PP7435_Chr4-0629 and PP7435_Chr4-1013 are clearly expressed (Table S2, Supporting Information), which indicates that the expression of these genes in standard growth media is independent of their telomeric localization, contrary to *S. cerevisiae*.

*Komagataella phaffii* also possesses a putative Flo8 homolog (PP7435_Chr4-0252) that contains an N-terminal LisH domain similar to the *S. cerevisiae FLO* gene master regulator Flo8p. Homology to the *S. cerevisiae* Flo8p is low outside of the LisH domain (Fig. 1B), and in *K. phaffii* there is no homolog of the second *FLO* transcriptional activator Mss11p, which forms a heterodimer with Flo8p in *S. cerevisiae* (Gagiano *et al*. 2003). In contrast to *S. cerevisiae* Flo8p, the *K. phaffii* Flo8 contains two predicted nuclear localization signals (NLS) and a putative SSDP domain at position 568–585 based on amino acid sequence homology to fungal Flo8/Som homologs (Lin *et al*. 2015; Bui *et al*. 2019). As described for these fungal species, we conclude that *K. phaffii* Flo8 is also probably forming homodimers to act as a transcriptional activator. Accordingly, in a *K. phaffii* strain lacking PP7435_Chr4-0252/Flo8, surface growth was much less pronounced and also pseudohyphal growth at slow growth rates was absent (Rebnegger *et al*. 2016).

### *Komagataella* *phaffii* CBS7435 forms pseudohyphae and displays surface adherence only at slow growth rates in glucose-limited chemostats but not during cultivation under glucose/nitrogen limitation or in presence of fusel alcohols

Reported triggers of pseudohyphal growth in *S. cerevisiae* are glucose or nitrogen limitation as well as the presence of fusel alcohols such as butanol or isoamyl alcohol (Gimeno *et al*. 1992; Dickinson 1996; Cullen and Sprague 2000; Lorenz *et al*. 2000). To investigate whether these conditions provoke the same response in *K. phaffii*, we exposed *K. phaffii* CBS7435 as well as *S. cerevisiae* Σ1278b (serving as a control) to such triggers and analyzed their morphology by microscopy.

We observed that cultivation on YP agar (without glucose) was unable to induce pseudohyphae formation in both *S. cerevisiae* and *K. phaffii* (Figure S1A, Supporting Information). Cultivation on minimal agar medium containing 24 mM ammonia induced pseudohyphal growth in *S. cerevisiae* but not in *K. phaffii*. Furthermore, when grown on minimal agar where the nitrogen content was severely reduced (50 µM ammonia) *K. phaffii* still did not display pseudohyphae formation, while the *S. cerevisiae* strain was unable to grow under these conditions at all (Figure S1B, Supporting Information). Additionally, when cultivated in the presence of butanol or isoamyl alcohol, *K. phaffii* again did not display any pseudohyphae formation contrary to *S. cerevisiae* (Figure S1C and D, Supporting Information). Since these observations indicated that the conditions that trigger pseudohyphal growth in *S. cerevisiae* are unable to provoke a similar response in *K. phaffii*, we based further experiments on the conditions where pseudohyphal growth was observed before, namely at low dilution rates in glucose-limited chemostats (Rebnegger *et al*. 2014).

**Figure 3.**
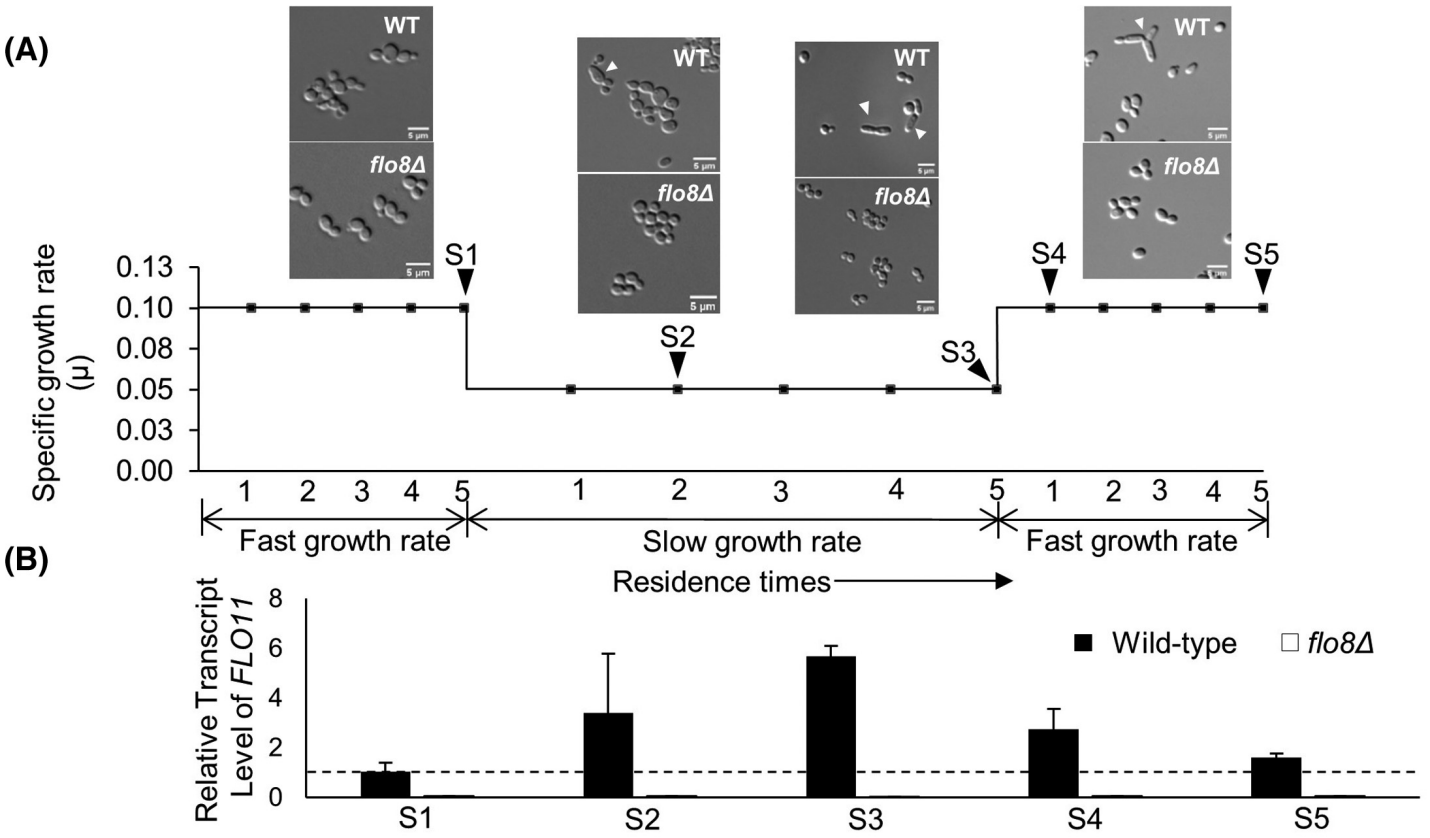
Cultivation at low dilution rate (corresponding to *µ* = 0.05 h^−1^) in glucose-limited chemostat triggers pseudohyphal growth in *K. phaffii*, which seems to be connected to the expression of *FLO11*. **(A)** Growth rate profile of the glucose-limited chemostat experiments, sampling time points (S1–S5) and cell morphology of the wild-type and the *flo8Δ* strains.**(B)** The plot shows the transcript level of *FLO11* in the wild-type and the *flo8Δ* strains, measured by qRT-PCR, relative to the reference sample S1 in the wild type.

We ran a glucose-limited chemostat first at a high dilution rate (corresponding to *µ* = 0.1 h^− 1^) for ∼5 residence times (50 h) corresponding to ∼7 generations, which serves as a control setpoint. Here, residence time refers to the amount of time taken to change the entire volume of the chemostat. At this point all the cells had an ovoid shape (S1 in Fig. 3A). Then the chemostat was switched to a low dilution rate (*µ* = 0.05 h^−1^). At this condition, after ∼2 residence times (corresponding to 3 generations), some cells in the population started to look more elongated (S2 in Fig. 3A). With increasing number of residence times at this low dilution rate, the effect became more pronounced. Additionally, some cells adopted a branched pseudohyphal appearance (S3 in Fig. 3A). Upon switching to the low dilution rate, cells also started growing on the surface of the bioreactors, which became even more pronounced with increasing number of residence times (Figure S2, Supporting Information). Strikingly, when the dilution rate was switched back to *µ* = 0.1 h^−1^, ∼17% of the cells in the population still exhibited an elongated or pseudohyphal phenotype even after 7 generations/5 residence times (S4 & S5 in Fig. 3A). This is striking, as at S5 over 99% of the cells never experienced the slow pseudohyphae-triggering growth rate (*µ* = 0.05 h^−1^) thus indicating that pseudohyphae formation upon slow growth in *K. phaffii* is a heritable phenotype.

### Pseudohyphal growth of *K. phaffii* is dependent on the presence of Flo8

As mentioned before, the *S. cerevisiae* laboratory strain S288C does not show any adhesion-related phenotype because of a single nonsense mutation in *FLO8* (Liu *et al*. 1996). To confirm that the pseudohyphal phenotype in *K. phaffii* is also dependent on the putative Flo8 homolog, we cultivated the *K. phaffii flo8Δ* strain (Gasser *et al*. 2015) at the same conditions as the wild type. In contrast to the wild-type strain, the *flo8Δ* strain showed no pseudohyphal phenotype and only very reduced surface growth (Fig. 3A; Figure S2, Supporting Information). Hence the samples from the *flo8Δ* cultivations were used as controls for further analysis.

For ease of understanding, henceforth the chemostat samples will be referred to using their sample names as given in Fig. 3A (S1, S2, S3, etc.).

### *FLO11* is induced in conditions of pseudohyphal growth, but *FLO11* deletion does not prevent pseudohyphae formation in *K. phaffii*

In *S. cerevisiae*, expression of *FLO11* is required for pseudohyphal growth and biofilm formation (Rupp *et al*. 1999; Cullen and Sprague 2012) and strains lacking the *FLO11* gene are unable to form pseudohyphae (Lo and Dranginis 1998). To verify whether the same gene also plays a crucial role in conferring the pseudohyphal phenotype in *K. phaffii*, we analyzed the expression profile of *FLO11* during different stages of the chemostat cultivations by employing quantitative real-time PCR (qRT-PCR) (Fig. 3B). Compared to the reference sample S1 at 0.1 h^−1^, *FLO11* expression significantly increased at S2 and S3, concurrent with the induction of pseudohyphal growth. Consistent with our observation that with an increasing number of residence times at the low dilution rate the pseudohyphal phenotype became more pronounced, expression of *FLO11* also got stronger. Upon switching the dilution rate back to 0.1 h^−1^ (sample S4), the expression of *FLO11* quickly dropped. However, even after five residence times (sample S5), the *FLO11* expression remained 1.5-fold higher compared to S1 (*P*-value < 0.05). In the *flo8Δ* strain, the *FLO11* expression remained below detection level, indicating that activation of the gene is under control of Flo8.

**Figure 4.**
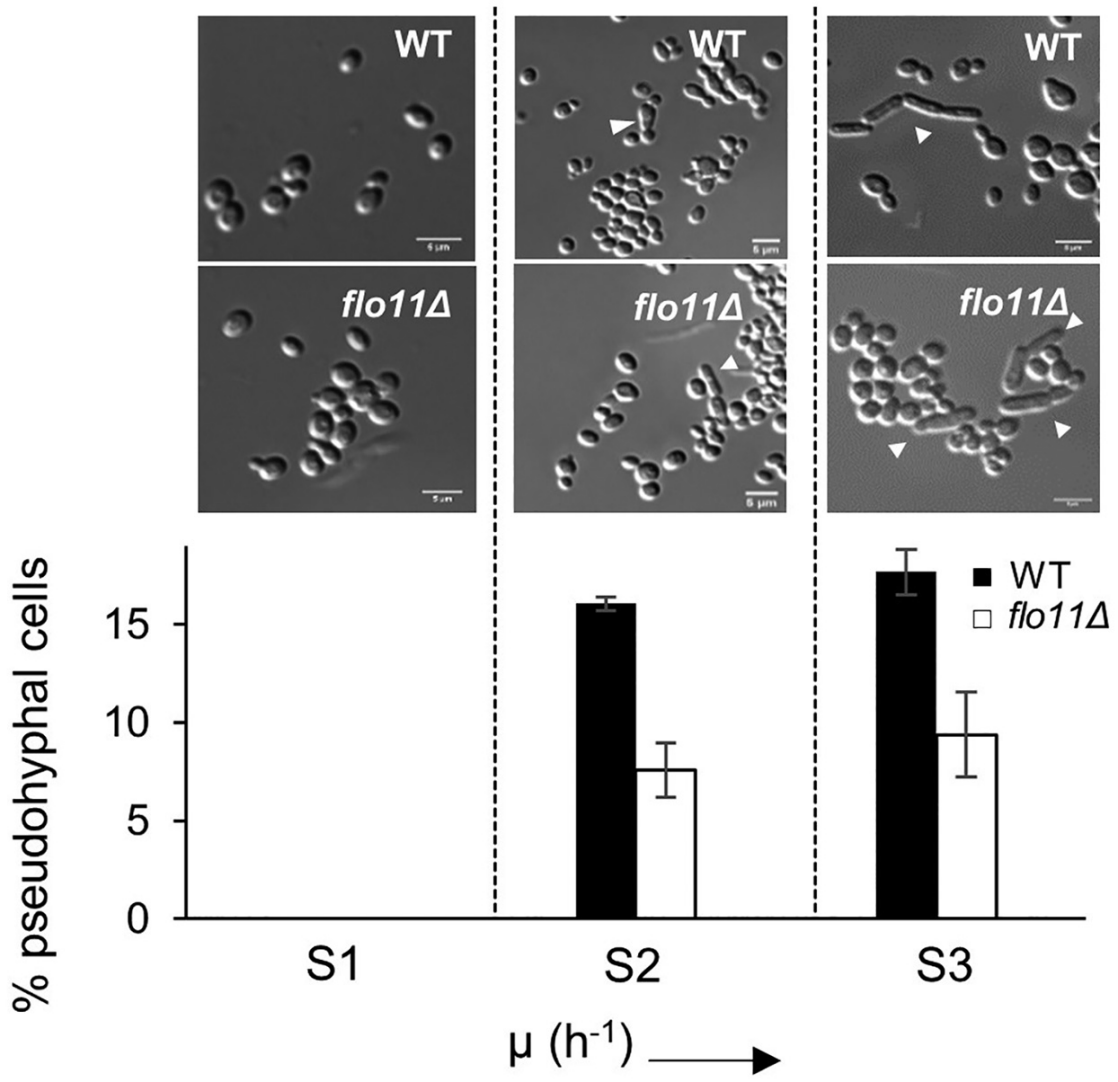
Microscope pictures of both the wild-type and the *flo11Δ* strains at different sampling time points. The graph shows the percentage of pseudohyphal cells in the population for both the wild-type and the *flo11Δ* strains (*n *= 3).

Based on the quantitative PCR results, we considered the possibility that, like in *S. cerevisiae*, *FLO11* is also the sole gene responsible for conferring the pseudohyphal phenotype in *K. phaffii*. To verify this, we constructed a *flo11Δ* strain and cultivated it in biological duplicates applying the same chemostat cultivation strategy as described before. As a control, we simultaneously cultivated the wild-type strain. However, we observed that unlike in *S. cerevisiae*, deleting *FLO11* did not inhibit pseudohyphal growth, although a cell count to compare the percentage of pseudohyphal (elongated) to non-pseudohyphal (ovoid) cells in the wild-type and the *flo11Δ* strains revealed that deletion of *FLO11* impairs pseudohyphal growth (Fig. 4). After five residence times at a dilution rate of 0.05 h^−1^, ∼17% of the wild-type population showed an elongated or pseudohyphal phenotype, while only 7.5% of the *flo11Δ* population switched to such a phenotype (Fig. 4) revealing that unlike in *S. cerevisiae*, *FLO11* is not the only player in conferring the pseudohyphal phenotype in *K. phaffii*.

### Quantification of mRNA levels of *FLO* genes by qRT-PCR and RNA-Seq

To check the expression patterns of the other *FLO* genes at fast and slow growth, we measured their transcript levels at the different growth rates in the wild-type as well as the *flo8Δ* strains by qRT-PCR and RNA-Seq.

**Figure 5.**
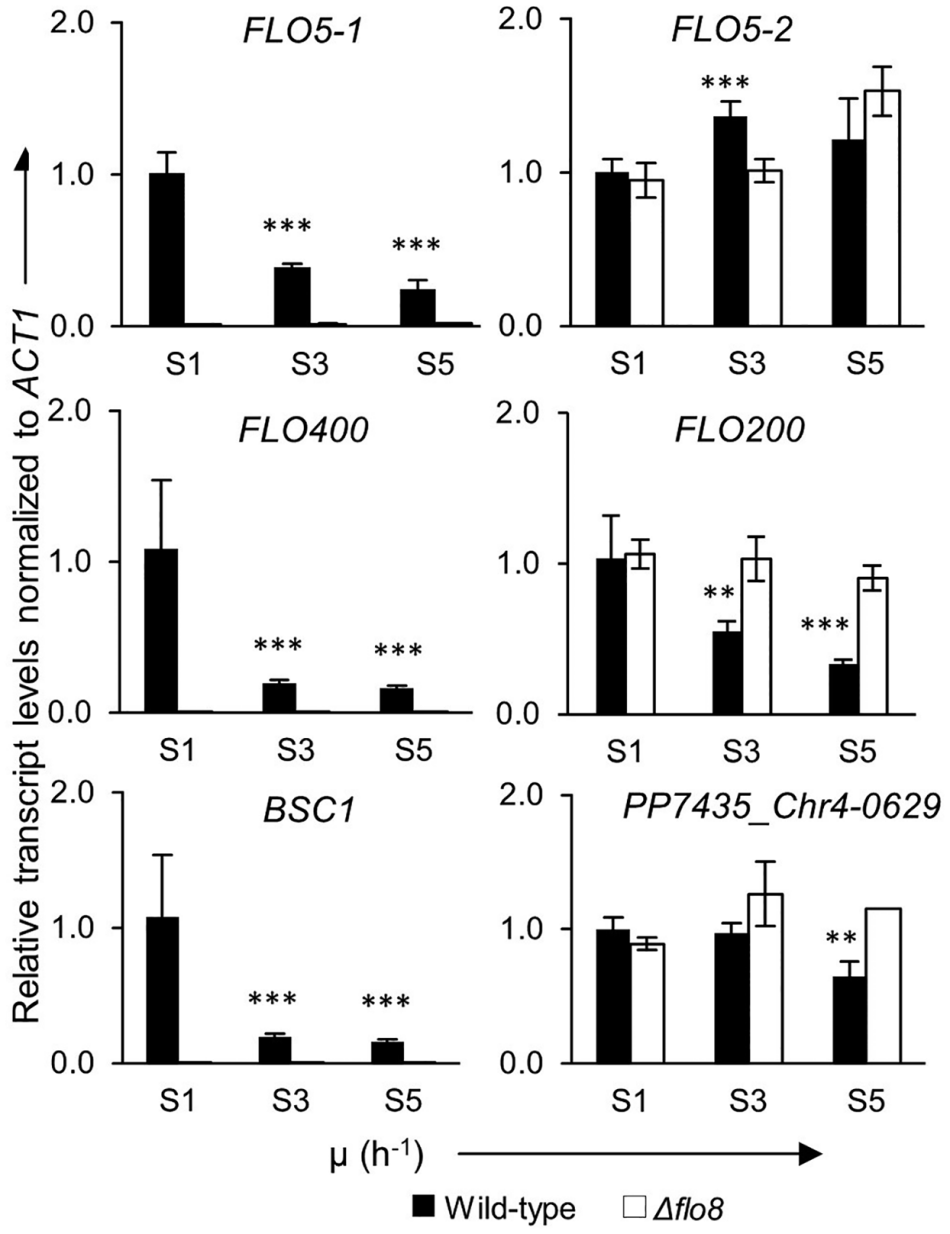
qRT-PCR data showing expression pattern of several *FLO* genes relative to the reference sample (wild type in S1). Asterisks above the bars denote statistical significance of gene expression levels in comparison with the reference sample (*P*-values calculated by Student's*t*-test; **P-value < 0.01 and ***P-value < 0.001).

qRT-PCR data showed that out of the analyzed genes, *BSC1*, *FLO5-1* and *FLO400* seemed to be under the control of Flo8 and showed no expression in the *flo8Δ* strain, while *FLO5-2, FLO200* and PP7435_Chr4-0629 were unaffected by the lack of Flo8 (Fig. 5). Four of the genes, *BSC1*, *FLO5-1*, *FLO200* and *FLO400* were downregulated at S3 in comparison to the reference sample S1. These genes stayed repressed even at S5. Considering that >99% of the cells present at S5 never experienced the slow pseudohyphae-triggering growth rate, it is speculated that these genes underwent stable repression.

Next, RNA-Seq was performed on the samples collected during the chemostats of the wild-type at S1, S3 and S5 and the *flo8Δ* strains at S1 and S3. Since the pseudohyphal phenotype is observed only in the wild-type strain but not in *flo8Δ*, we first looked at the genes that are differentially regulated between these strains. As mentioned in Materials and methods, transcripts with a log_2_ fold-change below −1 and above 1, combined with an adjusted *P*-value < 0.05 were considered to be significant. Using these criteria, it was seen that 107 genes were upregulated in the wild-type strain compared to the *flo8Δ* strain (Table S3, Supporting Information). Among the *FLO* genes this included *FLO11*, *FLO5-1*, *FLO400* and PP7435_Chr3-1237 (Table 2). On the other hand, only 21 genes were downregulated in the wild-type strain compared to the *flo8Δ* strain (Table S3, Supporting Information). Analyzing the Gene Ontology terms enriched in this list of differentially regulated genes using the open source software GO::TermFinder (Ashburner *et al*. 2000; Boyle *et al*. 2004), we found that most of them are related to cellular response to pheromone, reproductive processes and multi-organism processes. Interestingly, we also found a big group of unannotated genes (63 genes) in this list, which are not characterized so far. The enriched GO terms and the genes belonging to these GO terms are listed in Table S4 (Supporting Information).

**Table 2.** RNA-Seq data of all *FLO* genes showing the average transcripts per million (tpm) values of the *FLO* genes.

	Wild type *µ* = 0.1 h^−1^	Wild type *µ* = 0.05 h^−1^	Wild type *µ* = 0.1 h^−1^	*flo8Δ* *µ* = 0.1 h^−1^	*flo8Δ* *µ* = 0.05 h^−1^
Gene name	S1	S3	S5	S1	S3
*FLO11*	6.5	58.8	18.1	0.4	0.3
*FLO5-1*	2156.4	666.3	419.5	47.5	47.9
*FLO400*	3431.2	1776.4	1029.1	339.4	113.5
*BSC1*	4.8	4.4	6.1	6.3	4.2
*FLO5-2*	16.4	12.0	10.7	14.9	12.7
*FLO100*	0.3	5.3	33.7	0.2	0.0
*FLO200*	8.1	5.1	5.7	9.7	6.3
*FLO300*	8.0	42.3	161.0	9.3	25.6
PP7435_Chr1-2104	2942.1	3308.8	3697.5	3977.6	3642.0
PP7435_Chr3-1237	36.0	13.1	17.7	3.1	7.9
PP7435_Chr2-0004	10.9	8.1	7.4	9.9	8.2
PP7435_Chr4-1013	16.3	13.8	14.5	17.8	12.9
PP7435_Chr4-0629	74.5	73.7	67.4	89.6	86.3
*ACT1*	1726.9	1601.2	1616.6	1704.7	1483.7

We then looked specifically into the RNA-Seq data of the *FLO* genes in the wild-type strain. Table 2 shows the average transcripts per million (tpm) of the three biological replicates of each sample. The data for *ACT1* is also given for comparison. *FLO400*, *FLO5-1* and PP7435_Chr1-2104 were expressed at levels even higher than that of *ACT1*. While the expression of PP7435_Chr1-2104 did not undergo significant variation at the different stages of the chemostat cultivation, the expression of *FLO400* and *FLO5-1* changed upon switching to the slow growth rate. The highest expression levels for these two genes was reached initially at S1. At S3, both genes were repressed and remained in their repressed state even after switching back to the fast growth rate (S5) confirming the pattern of gene expression that we observed in the qRT-PCR data. PP7435_Chr3-1237 follows a similar expression and downregulation pattern as *FLO400* and *FLO5-1* but its expression level, even at S1, is much lower.

*FLO11* reached its highest expression during the slow growth rate, but even then, the expression level was much lower than the highest expression levels of *FLO400* and *FLO5-1*. The expression level of *FLO11* was again downregulated after the growth rate was switched back, but remained >2-fold higher in S5 than in S1, which reflected the pattern shown by the qRT-PCR data. In the *flo8Δ* strain the expression of *FLO400*, *FLO5-1*, *FLO11* and PP7435_Chr3-1237 genes was highly hampered, confirming that they are under the regulation of Flo8. PP7435_Chr4-0629 was stably expressed at a low level independent of the dilution rate. Even though expressed at lower levels compared to *FLO400* and *FLO5-1*, *FLO300* seems to be upregulated at the slow growth rate and shows even higher expression upon switching back to the fast growth rate, however, this expression pattern is similar in the *flo8Δ* strain.

We additionally created three different reporter strains expressing eGFP-tagged versions of Flo11, Flo400 and Flo5-1, respectively, and carried out similar glucose-limited chemostats with these strains. The purpose of these reporter strains was to follow the change in expression pattern of these proteins and to find out if these proteins are expressed in the entire population or only in a subpopulation, since pseudohyphal cells only represent a fraction of the population. As expected, both Flo400-eGFP and Flo5-1-eGFP were detected only in the S1 sample. Additionally, while Flo400-eGFP is produced in all the cells of the population, Flo5-1-eGFP was expressed only in a fraction of cells, which was ∼11% of the population (Figure S3, Supporting Information). Even though a similar N-terminal internal GFP tagging of Flo11 has been described in *S. cerevisiae* (Lo and Dranginis 1996), unfortunately the Flo11-eGFP expressing *K. phaffii* strain showed no observable fluorescence, which was most probably due to the general low expression levels of this protein.

### FAIRE-Seq analysis indicates involvement of chromatin modulation in the regulation of *FLO5-1* and *FLO400*

It has been reported that in *S. cerevisiae* some of the members of the *FLO* gene family are epigenetically silenced (Halme *et al*. 2004). The most well studied member in that regard is *FLO11*. The *FLO11* promoter, one of the largest described in *S. cerevisiae* with a size of around 3.5 kb, has binding sites for many different regulators and its expression is controlled by multiple levels of conventional and epigenetic regulation (Madhani and Fink 1997; Bumgarner *et al*. 2009; Octavio *et al*. 2009). The epigenetic regulation of *FLO11* involves chromatin modulation via histone modifications and nucleosome remodeling via the Rpd3L and Swi/Snf complex (Barrales *et al*. 2012). To investigate if and how changes in chromatin accessibility occur at a genome-wide level of fast and slow growing *K. phaffii*, we carried out Formaldehyde Assisted Isolation of Regulatory Elements (FAIRE-Seq) analysis (Giresi and Lieb 2009) of samples S1, S3 and S5. FAIRE-Seq analysis allows for the identification of open (nucleosome-free) chromatin regions, which include regulatory regions, at a genome-wide level during each condition.

Peaks in the FAIRE-Seq data correspond to open chromatin or nucleosome-free regions. Upon comparison and visualization of FAIRE-seq peaks between S1, S3 and S5 using the genome browser IGB, four different patterns were observed, namely, peaks that were detected at the same level in S1, S3 and S5 (indicating no chromatin modulation), peaks that were detected only at S3 (indicating open chromatin only at slow growth rate) and peaks that underwent stable changes between the samples S1 and S3, and remained in the same state in S5. These peaks were either detected in both S3 and S5 but not in S1 (Category I), or only detected in S1 but not in S3 and S5 (Category II). Screenshots of IGB showing representative chromosome regions with the different peak patterns are shown in Figure S4A–D (Supporting Information).

**Figure 6.**
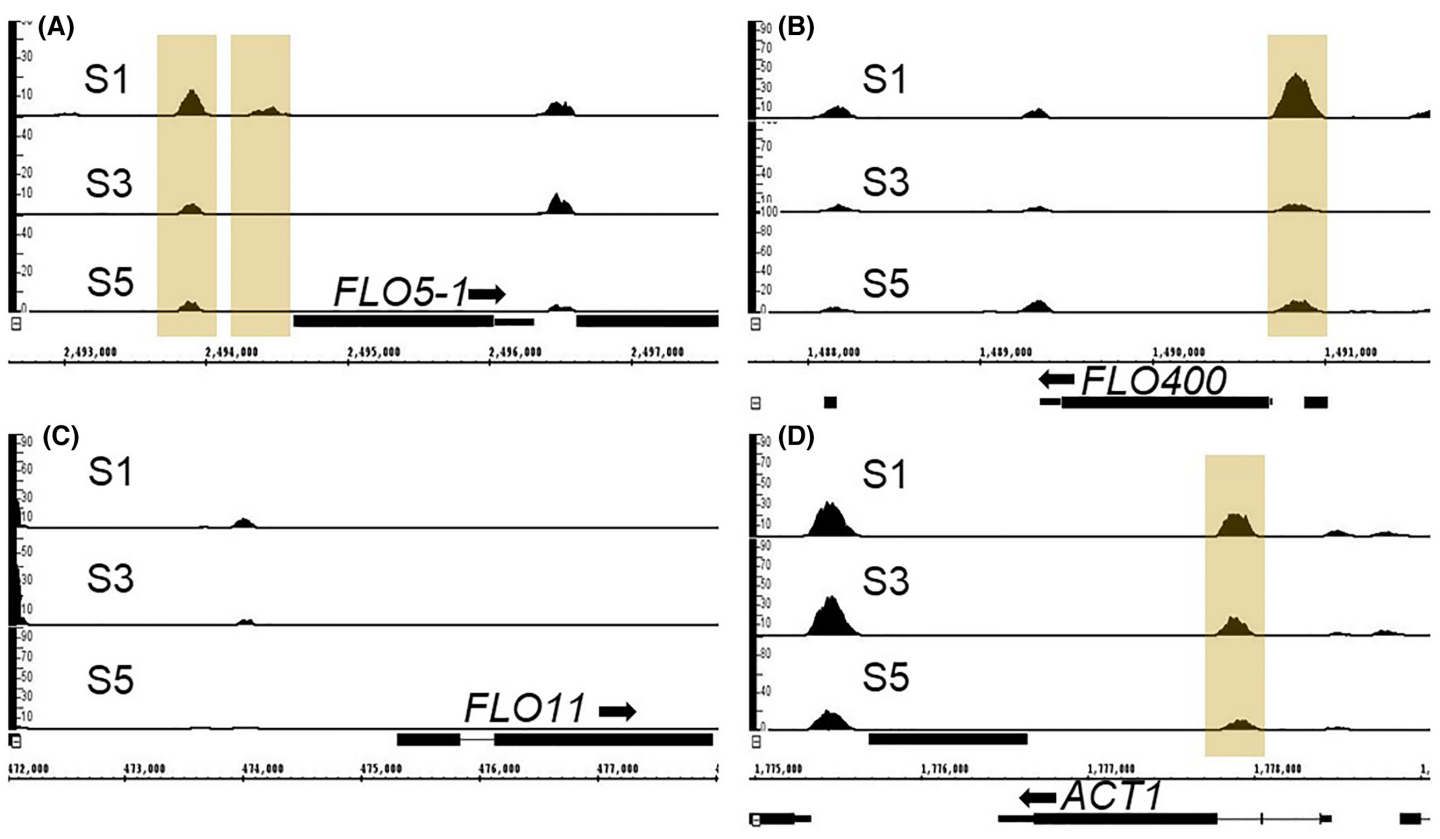
Open chromatin regions identified in the proximity of *FLO* genes by FAIRE-Seq analysis. Screenshot from IGB showing differential peaks upstream of *FLO5-1* and *FLO400* (highlighted in yellow); such statistically differential peaks are not observed in proximity of *FLO11*. *ACT1* is included here as control where an upstream open region is detected in all the three samples.

Regions corresponding to stable changes occurring in the chromatin after switching to the slow growth rate and returning to the fast growth rate (S1 vs S3 & S5) according to the FAIRE-Seq data were further analyzed. As explained above, Category I includes regions that were stably closed (nucleosome-bound) upon switching to the slow growth rate, while Category II contains regions that were stably opened (nucleosome-free) upon switching to the slow growth rate (Table S5 and Figure S4E and F, Supporting Information). The number of peaks in category I was much lower (around 25) compared to category II (around 350). In category I, peaks upstream of two flocculin genes, namely, *FLO400* and *FLO5-1* were detected, which means, the peaks upstream of these genes were detected only in sample S1 but not in S3 or S5, which indicate that the regions upstream of *FLO400* and *FLO5-1* undergo stable changes upon exposure to slow growth rate conditions. Screenshots of the MACS peak calling data for these regions as visualized on IGB for the wild-type sample are shown in Fig. 6A and B (Stein *et al*. 2002; Zhang *et al*. 2008). This is in accordance with the changes in transcript levels of these two genes as seen from the qRT-PCR and RNA-Seq data where the expression levels of both the genes are significantly downregulated in S3 and S5 (*P*-value < 10^−6^) compared to S1. Other than *FLO400* and *FLO5-1*, no peaks were identified in the proximity of *FLO11* (Fig. 6C) or any of the other *FLO* genes.

The regions in category II, even though being many more than category I, did not yield any significantly enriched GO terms. We looked at the genes that were proximal to these regions to specifically find genes that might be connected to the pseudohyphal phenotype. While we did find some genes like *MSB2*, *GTR2*, *MIT1*, *RSR1*, *KSS1* and *MIG1-1* that are connected to the regulation of filamentous growth in *S. cerevisiae*, on correlating these genes with the RNA-Seq data no significant upregulation of these genes in S3 compared to S1 was seen.

Finally, the FAIRE-Seq data of the *flo8Δ* strain was compared with that of the wild-type strain at each time point but no significant differences between the open chromatin regions in the wild-type and the *flo8Δ* strains were observed. This indicates that Flo8 does not play any role in the modification of chromatin structure.

### Deletion of *FLO400* or *FLO5-1* prevents pseudohyphal growth and expression of *FLO11* at slow growth rates

**Figure 7.**
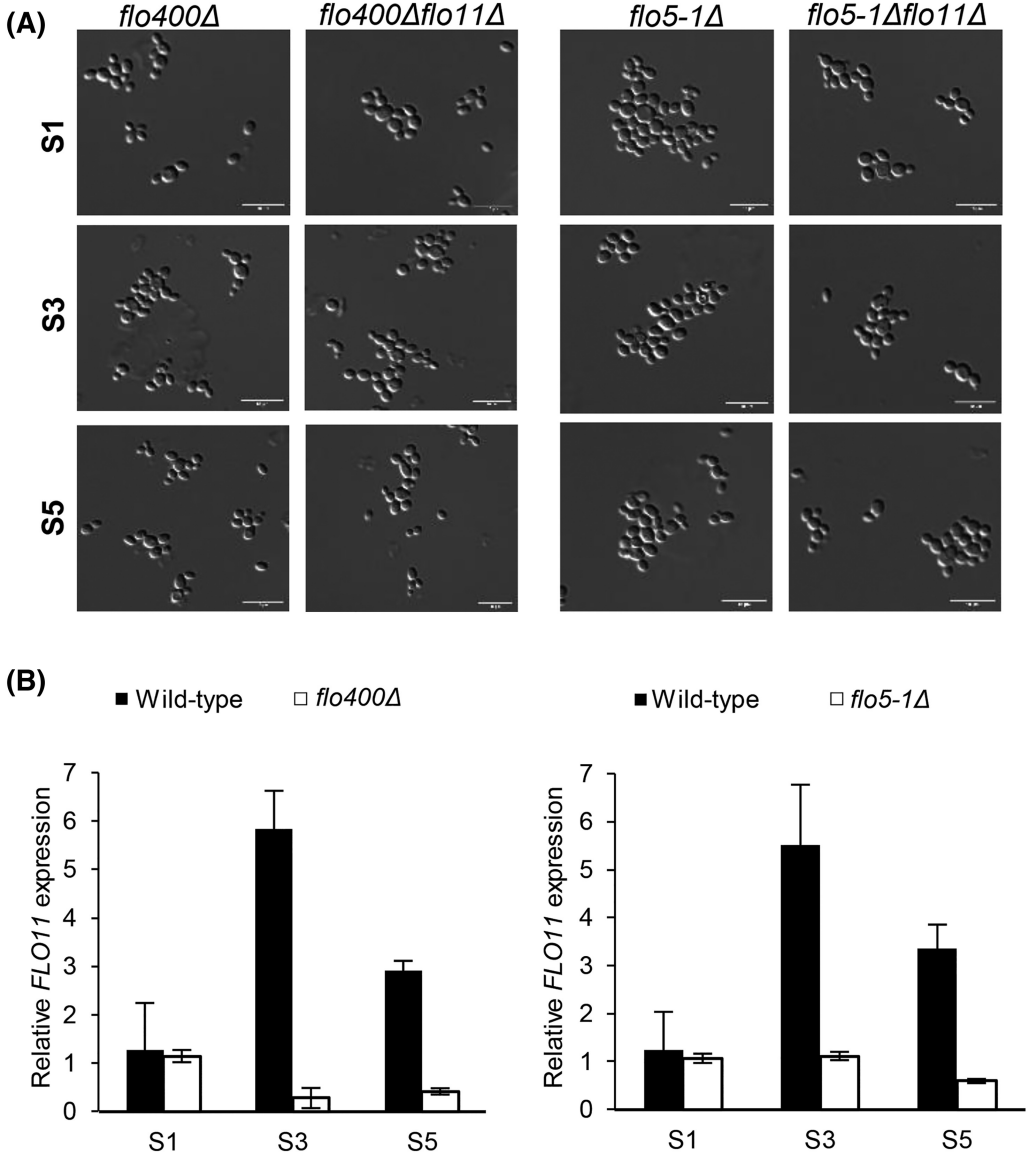
*FLO5-1* and *FLO400* are major players involved in the regulation of pseudohyphal growth. **(A)**Microscope pictures of *flo5-1Δ* and *flo400Δ* strains in both wild-type and *flo11Δ* backgrounds at sampling time points S1, S3 and S5. **(B)** qRT-PCR data for *FLO11* expression in the *flo400Δ* (right) and *flo5-1Δ* (left) strains relative to the reference sample at S1.

Based on the regulation patterns observed for *FLO400* and *FLO5-1* during cultivation at fast and slow growth rates, the FAIRE-Seq data and the fact that both of them are controlled by the Flo8 transcription factor, they were considered as possible candidates that participate in the pseudohyphal phenotype. Thus, we generated deletion strains of these two genes individually, both in the wild-type as well as in the *flo11Δ* strains background. Again, we performed chemostat runs as described before with these four strains (*flo5-1*Δ, *flo5-1Δ flo11Δ, flo400Δ*, *flo400Δ flo11Δ*), each in biological duplicates. We observed that in both the *flo5-1Δ* and *flo400Δ* strains pseudohyphae formation was absent, in the wild-type as well as in the *flo11Δ* background (Fig. 7A). Strikingly, qRT-PCR for checking *FLO11* expression in the *flo400Δ* and *flo5-1Δ* strains revealed that *FLO11* transcript levels were strongly reduced in both the deletion strains, which indicates that the presence of both Flo5-1 and Flo400 is necessary for the expression of *FLO11* (Fig. 7B).

## DISCUSSION

### Pseudohyphal growth in *K. phaffii* is triggered under different environmental conditions compared with *S. cerevisiae*

Our data indicate that pseudohyphal growth behavior in *K. phaffii* differs substantially from that in *S. cerevisiae* including the specific trigger for transition into this phenotype as well as its regulation. *Saccharomyces cerevisiae* exhibits pseudohyphal growth in both its diploid and haploid form in response to nutrient limitation or presence of fusel alcohols. In contrast, *K. phaffii*, which preferentially exists as a haploid, shows no switch to the pseudohyphal phenotype when grown on glucose- or nitrogen-limited agar plates or when cultivated in the presence of fusel alcohols (Figure S1, Supporting Information). N-depleted *K. phaffii* were reported to rather undergo mating (Heistinger *et al*. 2018). Until now, the only condition where *K. phaffii* has been shown to transition to pseudohyphal growth is at slow growth rates in glucose-limited chemostats, first reported in the study by Rebnegger *et al*. (2014) and subsequently confirmed in our experiments. The initiation of the phenotype in *K. phaffii* seems to be tightly regulated as the appearance of the first elongated cells did not occur immediately upon switching to the slow growth rate conditions. Instead it required at least 2 residence times—corresponding to ∼3 generations—until the first elongated cells appeared, although after its initiation the phenotype became stronger with increasing residence times at the slow growth rate (Fig. 3A). It could be speculated that unlike in *S. cerevisiae, K. phaffii* requires prolonged exposure to glucose-limited conditions and/or more severe limitation to initiate a switch in phenotype. This is consistent with the fact that as a Crabtree-negative yeast, *K. phaffii* has a high-affinity glucose uptake system with a glucose saturation constant (K_s_) in the range of 9.7 µM at high growth rates (Prielhofer *et al*. 2013), which is much lower than those of the high affinity hexose transporters of *S. cerevisiae* where the K_s_ is around 1 mM (Boles and Hollenberg 1997; Reifenberger *et al*. 1997).

Apart from pseudohyphal growth, slow growth rate conditions in the glucose-limited chemostat also lead to surface adherence of cells indicating that such prolonged exposure to glucose limitation might also trigger other cell differentiations, thus leading to the formation of different subpopulations of cells, namely, ovoid, pseudohyphal and surface adhering cells. This is not surprising since it is known from *S. cerevisiae* and other yeasts that environmental stress can trigger multiple cellular differentiations to maximize chances of survival. It has already been contemplated that the pseudohyphal phenotype provides an advantage in nutrient-limiting conditions because the elongated shape enables yeasts to access distant nutrients more efficiently (Honigberg 2016). Additionally, the higher surface-to-volume ratio of pseudohyphal cells provides more space for absorption of nutrients (Adams *et al*. 1985). Adherence to a foreign surface provides the cells with the capability to invade the surface to forage for nutrients (Verstrepen and Klis 2006).

The control *flo8Δ* strain could not initiate pseudohyphal growth and also showed highly reduced surface growth in the bioreactor, which confirmed that, like in *S. cerevisiae*, in *K. phaffii* Flo8 is also an important player in the regulation of adhesion-related phenotypes. The non-adhering phenotype of *flo8Δ* might also provide a beneficial performance in industrial bioprocesses.

### *FLO11* is not the only major gene responsible for conferring pseudohyphal phenotype in *K. phaffii*

qRT-PCR analysis showed that the initiation of expression of *FLO11* in *K. phaffii* is in accordance with the appearance and persistence of the elongated/pseudohyphal cells. This implication was enforced by the observation that in the non-transitioning *flo8Δ* strain *FLO11* expression cannot be detected (Fig. 3B and Table 2). Contrary to *S. cerevisiae flo11Δ* strains, the *K. phaffii flo11Δ* strain could still switch to pseudohyphal growth, even though the number of cells switching to this phenotype was lower than in the wild-type strain (Fig. 4). So, it can be said that while in *K. phaffii FLO11* does play a role in pseudohyphae formation, it is not the only player and clearly there is the involvement of other gene(s).

### *FLO400* and *FLO5-1* are crucial players required for initiation of pseudohyphal differentiation in *K. phaffii*

To identify which other gene(s) might be involved, we investigated the expression patterns of other *FLO* genes under the same conditions. This revealed the genes *FLO400* and *FLO5-1* (Fig. 5; Table 2), which are expressed initially at the fast growth rate, but undergo stable repression upon switching to the slow growth rate. Both genes were downregulated in the *flo8Δ* strain. Reporter strains expressing Flo400-eGFP and Flo5-1-eGFP confirmed this expression pattern, and showed that Flo400 was expressed in all the cells of the population, while Flo5-1 was expressed in only a subpopulation of cells (Figure S3, Supporting Information). It remains to be discovered whether the cells expressing Flo5-1 in S1 are those that transition to pseudohyphal growth in S3.

Since we found indications of stable repression of *FLO5-1* and *FLO400* triggered by prolonged glucose limitation, we performed FAIRE-Seq analysis to investigate the involvement of chromatin organization. Upstream open chromatin regions are usually associated with the proficiency of transcriptional activation of these genes. Open chromatin regions were identified upstream of *FLO5-1* and *FLO400* at the initial fast growth rate (S1) in accordance to their high expression levels in this condition. After switching to the slow growth rate, no upstream peaks were seen anymore at S3 and S5, indicating that these chromatin regions underwent stable conformational changes, which could be a factor regulating the expression pattern observed for these two genes (Fig. 6A and B). It was shown that these chromatin modulations occurred independently whether Flo8 was present or not. Thus, the question arises if these stable changes are a result of histone modifications in these regions leading to epigenetic silencing of these genes as reported for *FLO* genes in *S. cerevisiae*. This can be verified by analyzing these regions further, for example, using enChIP-MS (Fujita and Fujii 2013) that might help to identify proteins interacting with these regions.

Absence of *FLO400* or *FLO5-1* was sufficient for eliminating pseudohyphae formation. Unlike the *flo8Δ* strain though, these knock-out strains still displayed surface adherence similar to the wild type. So, while not being involved in other morphogenic behaviors such as surface adherence, both *FLO400* and *FLO5-1* seem to play a major role in the transition to pseudohyphal growth. Further work should aim at verifying if epigenetics and epigenetic factors are indeed involved in the observed chromatin modulations.

### *Flo400* and *Flo5-1*: actors upstream of *FLO11*?

Absence of *FLO400* or *FLO5-1* prevented transition to pseudohyphal growth and was found to correlate to lack of *FLO11* induction at slow growth rate. Therefore, it is logical to assume that *FLO400* and *FLO5-1* act in concert with *FLO11* to initiate pseudohyphal growth in a subpopulation of *K. phaffii*. In fact, our observations indicate that *FLO400* and *FLO5-1* possibly act upstream in the signal cascade leading to the expression of *FLO11* (Fig. 7B) since the effect of deleting these two genes is much more pronounced than deleting *FLO11*. Although our experiments reveal new information about adhesion-related cellular differentiation in *K. phaffii*, involvement of chromatin modulation and novel players in the regulation mechanism of pseudohyphal differentiation, the mechanism by which Flo400, Flo5-1 and Flo11 interact still remains unclear and the roles of these proteins in pseudohyphal differentiation can only be speculated. While the expression pattern of *FLO400* and *FLO5-1* might seem contradictory to the observations in the knock-out strains in the first place, we assume that the presence of Flo400 and/or Flo5-1 is needed to allow for activation—but not maintenance—of *FLO11* expression. While such a regulation mechanism for *S. cerevisiae FLO11* is not known, it has been reported that two *S. cerevisiae* signaling mucins, Hkr2 and Msb2 that are homologous to Flo11 are shed from cells and act as osmosensors in the yeast HOG pathway (Tatebayashi *et al*. 2007; Vadaie *et al*. 2008) and both of these mucins have been reported to differentially activate the filamentous growth pathway (Pitoniak *et al*. 2009). However, while both Flo5-1 and Flo400 have threonine/proline rich internal repeat regions that are typical of mucins, unlike mucins neither of the two proteins has a transmembrane domain. Interestingly, during secretome analysis the Flo5-1 protein was found in the supernatants of *K. phaffii* cells in the later samples of glucose- or methanol-limited fed batch cultivations, where cells tend to adhere to the reactor surface as well (Burgard *et al*. 2020), indicating that a mechanism of cellular release might also act in *K. phaffii*.

**Figure 8.**
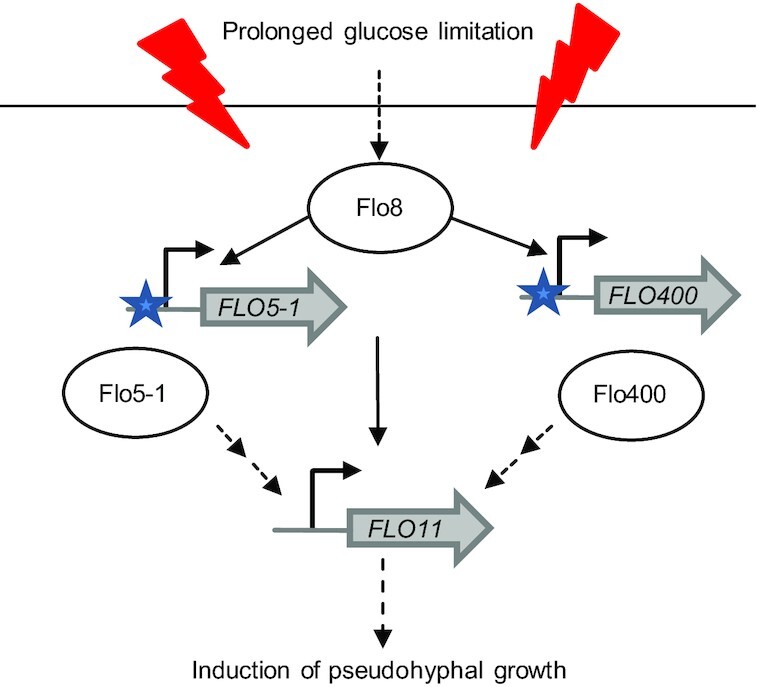
Scheme representing the proposed interplay of Flo8, Flo400, Flo5-1 and Flo11 in the cascade leading to pseudohyphal growth in *K. phaffii*.

Considering the essential role that both these genes play in pseudohyphae formation and *FLO11* expression, it could be speculated that Flo400 and Flo5-1 act as sensing proteins acting downstream of *FLO8* and upstream of *FLO11* in the signaling cascade leading to the formation of pseudohyphal cells (Fig. 8). It is interesting to note here that there is a rather large group of non-annotated genes that seem to be regulated by Flo8 (i.e. 90 out of 209 differentially regulated genes in *flo8Δ* compared to the wild-type strain at S3; Table S3, Supporting Information). As it is possible that this list includes components of the signal transduction pathway that leads to cellular differentiation in response to glucose limitation, characterization of these genes might help to provide further insight into the regulation of *FLO* genes and the pseudohyphal phenotype in the future.

## Supplementary Material

foaa044_Supplemental_File

## Data Availability

The raw RNA-Seq data, as well as the according gene expression results, were uploaded to NCBI GEO (Accession ID: GSE131106; Reviewer Token: wjwbgkmgbzqtxeh). For the FAIRE-Seq data, the raw data as well as the called peaks were uploaded to NCBI GEO in bed-format (Accession ID: GSE131290; Reviewer Token: apgrgyairzapdyl).

## References

[bib1] Adams J , Paquin C, Oeller PW et al. Physiological characterization of adaptive clones in evolving populations of the yeast, *Saccharomyces cerevisiae*. Genetics. 1985;110:173–85. 3891508 10.1093/genetics/110.2.173 PMC1202558

[bib2] Afgan E , Baker D, Batut B et al. The Galaxy platform for accessible, reproducible and collaborative biomedical analyses: 2018 update. Nucleic Acids Res. 2018;46:W537–44. 29790989 10.1093/nar/gky379 PMC6030816

[bib3] Andrews S , Fast QC. A quality control tool for high throughput sequence data. http://www.bioinformatics.babraham.ac.uk/projects/fastqc/2010, (28 January, 2018, date last accessed).

[bib4] Ashburner M , Ball CA, Blake JA et al. Gene ontology: tool for the unification of biology. The Gene Ontology Consortium. Nat Genet. 2000;25:25–9. 10802651 10.1038/75556 PMC3037419

[bib5] Ata Ö , Rebnegger C, Tatto NE et al. A single Gal4-like transcription factor activates the Crabtree effect in *Komagataella phaffii*. Nat Commun. 2018;9:4911. 30464212 10.1038/s41467-018-07430-4 PMC6249229

[bib6] Barrales RR , Korber P, Jimenez J et al. Chromatin modulation at the *FLO11* promoter of *Saccharomyces cerevisiae* by HDAC and Swi/Snf complexes. Genetics. 2012;191:791–803. 22542969 10.1534/genetics.112.140301 PMC3389975

[bib7] Biswas S , Van Dijck P, Datta A. Environmental sensing and signal transduction pathways regulating morphopathogenic determinants of *Candida albicans*. Microbiol Mol Biol Rev. 2007;71:348–76. 17554048 10.1128/MMBR.00009-06 PMC1899878

[bib8] Boles E , Hollenberg CP. The molecular genetics of hexose transport in yeasts. FEMS Microbiol Rev. 1997;21:85–111. 9299703 10.1111/j.1574-6976.1997.tb00346.x

[bib9] Boyle EI , Weng S, Gollub J et al. GO::TermFinder–open source software for accessing Gene Ontology information and finding significantly enriched Gene Ontology terms associated with a list of genes. Bioinformatics. 2004;20:3710–5. 15297299 10.1093/bioinformatics/bth456 PMC3037731

[bib10] Bray NL , Pimentel H, Melsted P et al. Near-optimal probabilistic RNA-seq quantification. Nat Biotechnol. 2016;34:525–7. 27043002 10.1038/nbt.3519

[bib11] Broad Institute . Picard Tools-By Broad Institute GitHub. http://broadinstitute.github.io/picard/ (22 January 2018, date last accessed), 2018.

[bib12] Bui TT , Harting R, Braus-Stromeyer SA et al. *Verticillium dahliae* transcription factors Som1 and Vta3 control microsclerotia formation and sequential steps of plant root penetration and colonisation to induce disease. New Phytol. 2019;221:2138–59. 30290010 10.1111/nph.15514

[bib13] Bumgarner SL , Dowell RD, Grisafi P et al. Toggle involving cis-interfering noncoding RNAs controls variegated gene expression in yeast. Proc Natl Acad Sci USA. 2009;106:18321–6. 19805129 10.1073/pnas.0909641106 PMC2775344

[bib14] Burgard J , Grünwald-Gruber C, Altmann F et al. The secretome of *Pichia pastoris* in fed-batch cultivations is largely independent of the carbon source but changes quantitatively over cultivation time. Microb Biotechnol. 2020;13:479–94. 31692260 10.1111/1751-7915.13499 PMC7017826

[bib15] Caro LH , Tettelin H, Vossen JH et al. *In silico* identification of glycosyl-phosphatidylinositol-anchored plasma-membrane and cell wall proteins of *Saccharomyces cerevisiae*. Yeast. 1997;13:1477–89. 9434352 10.1002/(SICI)1097-0061(199712)13:15<1477::AID-YEA184>3.0.CO;2-L

[bib16] Cullen PJ , Sprague GF. Glucose depletion causes haploid invasive growth in yeast. Proc Natl Acad Sci USA. 2000;97:13619–24. 11095711 10.1073/pnas.240345197 PMC17625

[bib17] Cullen PJ , Sprague GF. The regulation of filamentous growth in yeast. Genetics. 2012;190:23–49. 22219507 10.1534/genetics.111.127456 PMC3249369

[bib18] Dickinson JR . ‘Fusel’ alcohols induce hyphal-like extensions and pseudohyphal formation in yeasts. Microbiology. 1996;142:1391–7. 8704979 10.1099/13500872-142-6-1391

[bib19] Fairhead C , Llorente B, Denis F et al. New vectors for combinatorial deletions in yeast chromosomes and for gap-repair cloning using ‘split-marker’ recombination. Yeast. 1996;12:1439–57. 8948099 10.1002/(SICI)1097-0061(199611)12:14%3C1439::AID-YEA37%3E3.0.CO;2-O

[bib20] Fujita T , Fujii H. Efficient isolation of specific genomic regions and identification of associated proteins by engineered DNA-binding molecule-mediated chromatin immunoprecipitation (enChIP) using CRISPR. Biochem Biophys Res Commun. 2013;439:132–6. 23942116 10.1016/j.bbrc.2013.08.013

[bib21] Gagiano M , Bester M, van Dyk D et al. Mss11p is a transcription factor regulating pseudohyphal differentiation, invasive growth and starch metabolism in *Saccharomyces cerevisiae* in response to nutrient availability. Mol Microbiol. 2003;47:119–34. 12492858 10.1046/j.1365-2958.2003.03247.x

[bib22] Gasser B , Mattanovich D, Buchetics M. Recombinant host cell for expression proteins of interest: WO2015158808A2. https://patents.google.com/patent/WO2015158808A2/en (22 October 2015, date last accessed).

[bib23] Gasser B , Prielhofer R, Marx H et al. *Pichia pastoris*: protein production host and model organism for biomedical research. Future Microbiol. 2013;8:191–208. 23374125 10.2217/fmb.12.133

[bib24] Gassler T , Heistinger L, Mattanovich D et al. CRISPR/Cas9-mediated homology-directed genome editing in *Pichia pastoris*. Methods Mol Biol. 2019;1923:211–25. 30737742 10.1007/978-1-4939-9024-5_9

[bib25] Gentleman RC , Carey VJ, Bates DM et al. Bioconductor: open software development for computational biology and bioinformatics. Genome Biol. 2004;5:R80. 15461798 10.1186/gb-2004-5-10-r80 PMC545600

[bib26] Gimeno CJ , Ljungdahl PO, Styles CA et al. Unipolar cell divisions in the yeast *S. cerevisiae* lead to filamentous growth: regulation by starvation and RAS. Cell. 1992;68:1077–90. 1547504 10.1016/0092-8674(92)90079-r

[bib27] Giresi PG , Lieb JD. Isolation of active regulatory elements from eukaryotic chromatin using FAIRE (Formaldehyde Assisted Isolation of Regulatory Elements). Methods. 2009;48:233–9. 19303047 10.1016/j.ymeth.2009.03.003 PMC2710428

[bib28] Govender P , Domingo JL, Bester MC et al. Controlled expression of the dominant flocculation genes *FLO1*, *FLO5*, and *FLO11* in *Saccharomyces cerevisiae*. Appl Environ Microbiol. 2008;74:6041–52. 18708514 10.1128/AEM.00394-08 PMC2565957

[bib29] Guo B , Styles CA, Feng Q et al. A *Saccharomyces* gene family involved in invasive growth, cell-cell adhesion, and mating. Proc Natl Acad Sci USA. 2000;97:12158–63. 11027318 10.1073/pnas.220420397 PMC17311

[bib30] Halme A , Bumgarner S, Styles C et al. Genetic and epigenetic regulation of the *FLO* gene family generates cell-surface variation in yeast. Cell. 2004;116:405–15. 15016375 10.1016/s0092-8674(04)00118-7

[bib31] Heistinger L , Gasser B, Mattanovich D. Creation of stable heterothallic strains of *Komagataella phaffii* enables dissection of mating gene regulation. Mol Cell Biol. 2018;38:e00398–17. 29061733 10.1128/MCB.00398-17 PMC5748462

[bib32] Honigberg SM . Similar environments but diverse fates: responses of budding yeast to nutrient deprivation. Microb Cell. 2016;3:302–28. 27917388 10.15698/mic2016.08.516 PMC5134742

[bib33] Huber W , Carey VJ, Gentleman R et al. Orchestrating high-throughput genomic analysis with bioconductor. Nat Methods. 2015;12:115–21. 25633503 10.1038/nmeth.3252 PMC4509590

[bib34] Jaiswal D , Turniansky R, Green EM. Choose your own adventure: the role of histone modifications in yeast cell fate. J Mol Biol. 2017;429:1946–57. 27769718 10.1016/j.jmb.2016.10.018 PMC5395361

[bib35] Kersey PJ , Allen JE, Allot A et al. Ensembl Genomes 2018: an integrated omics infrastructure for non-vertebrate species. Nucleic Acids Res. 2018;46:D802–8. 29092050 10.1093/nar/gkx1011 PMC5753204

[bib36] Krueger F . Trim Galore! Babraham Bioinformatics. http://www.bioinformatics.babraham.ac.uk/projects/trim_galore/ (15 February 2017, date last accessed), 2012.

[bib37] Lambrechts MG , Bauer FF, Marmur J et al. Muc1, a mucin-like protein that is regulated by Mss10, is critical for pseudohyphal differentiation in yeast. Proc Natl Acad Sci USA. 1996;93:8419–24. 8710886 10.1073/pnas.93.16.8419 PMC38686

[bib38] Langmead B , Salzberg SL. Fast gapped-read alignment with Bowtie 2. Nat Methods. 2012;9:357–9. 22388286 10.1038/nmeth.1923 PMC3322381

[bib39] Li H , Handsaker B, Wysoker A et al. The Sequence Alignment/Map format and SAMtools. Bioinformatics. 2009;25:2078–9. 19505943 10.1093/bioinformatics/btp352 PMC2723002

[bib40] Lin CJ , Sasse C, Gerke J et al. Transcription factor SomA is required for adhesion, development and virulence of the human pathogen *Aspergillus fumigatus*. PLoS Pathog. 2015;11:e1005205. 26529322 10.1371/journal.ppat.1005205 PMC4631450

[bib41] Linder T , Gustafsson CM. Molecular phylogenetics of ascomycotal adhesins–a novel family of putative cell-surface adhesive proteins in fission yeasts. Fungal Genet Biol. 2008;45:485–97. 17870620 10.1016/j.fgb.2007.08.002

[bib42] Liu H , Styles CA, Fink GR. *Saccharomyces cerevisiae* S288C has a mutation in FLO8, a gene required for filamentous growth. Genetics. 1996;144:967–78. 8913742 10.1093/genetics/144.3.967 PMC1207636

[bib45] Lorenz MC , Cutler NS, Heitman J. Characterization of alcohol-induced filamentous growth in *Saccharomyces cerevisiae*. Mol Biol Cell. 2000;11:183–99. 10637301 10.1091/mbc.11.1.183 PMC14767

[bib46] Love MI , Huber W, Anders S. Moderated estimation of fold change and dispersion for RNA-seq data with DESeq2. Genome Biol. 2014;15:550. 25516281 10.1186/s13059-014-0550-8 PMC4302049

[bib43] Lo WS , Dranginis AM. FLO11, a yeast gene related to the *STA* genes, encodes a novel cell surface flocculin. J Bacteriol. 1996;178:7144–51. 8955395 10.1128/jb.178.24.7144-7151.1996 PMC178626

[bib44] Lo WS , Dranginis AM. The cell surface flocculin Flo11 is required for pseudohyphae formation and invasion by *Saccharomyces cerevisiae*. Mol Biol Cell. 1998;9:161–71. 9436998 10.1091/mbc.9.1.161 PMC25236

[bib47] Madhani HD , Fink GR. Combinatorial control required for the specificity of yeast MAPK signaling. Science. 1997;275:1314–7. 9036858 10.1126/science.275.5304.1314

[bib48] Marchler-Bauer A , Lu S, Anderson JB et al. CDD: a conserved domain database for the functional annotation of proteins. Nucleic Acids Res. 2011;39:D225–9. 21109532 10.1093/nar/gkq1189 PMC3013737

[bib49] Martin M . Cutadapt removes adapter sequences from high-throughput sequencing reads. EMBnetjournal. 2010;17:200.

[bib50] Marx H , Mattanovich D, Sauer M. Overexpression of the riboflavin biosynthetic pathway in *Pichia pastoris*. Microb Cell Fact. 2008;7:23. 18664246 10.1186/1475-2859-7-23 PMC2517057

[bib51] Moreno-García J , García-Martínez T, Mauricio JC et al. Yeast immobilization systems for alcoholic wine fermentations: actual trends and future perspectives. Front Microbiol. 2018;9:241. 29497415 10.3389/fmicb.2018.00241 PMC5819314

[bib52] Nagy PL , Cleary ML, Brown PO et al. Genomewide demarcation of RNA polymerase II transcription units revealed by physical fractionation of chromatin. Proc Natl Acad Sci USA. 2003;100:6364–9. 12750471 10.1073/pnas.1131966100 PMC164452

[bib53] Nicol JW , Helt GA, Blanchard SG et al. The integrated genome browser: free software for distribution and exploration of genome-scale datasets. Bioinformatics. 2009;25:2730–1. 19654113 10.1093/bioinformatics/btp472 PMC2759552

[bib54] Octavio LM , Gedeon K, Maheshri N. Epigenetic and conventional regulation is distributed among activators of *FLO11* allowing tuning of population-level heterogeneity in its expression. PLos Genet. 2009;5:e1000673. 19798446 10.1371/journal.pgen.1000673 PMC2745563

[bib55] Pitoniak A , Birkaya B, Dionne HM et al. The signaling mucins Msb2 and Hkr1 differentially regulate the filamentation mitogen-activated protein kinase pathway and contribute to a multimodal response. Mol Biol Cell. 2009;20:3101–14. 19439450 10.1091/mbc.E08-07-0760 PMC2704161

[bib56] Prielhofer R , Barrero JJ, Steuer S et al. GoldenPiCS: a Golden Gate-derived modular cloning system for applied synthetic biology in the yeast *Pichia pastoris*. BMC Syst Biol. 2017;11:123. 29221460 10.1186/s12918-017-0492-3 PMC5723102

[bib57] Prielhofer R , Maurer M, Klein J et al. Induction without methanol: novel regulated promoters enable high-level expression in *Pichia pastoris*. Microb Cell Fact. 2013;12:5. 23347568 10.1186/1475-2859-12-5 PMC3615954

[bib58] Rebnegger C , Graf AB, Valli M et al. In *Pichia pastoris*, growth rate regulates protein synthesis and secretion, mating and stress response. Biotechnol J. 2014;9:511–25. 24323948 10.1002/biot.201300334 PMC4162992

[bib59] Rebnegger C , Vos T, Graf AB et al. *Pichia pastoris* exhibits high viability and a low maintenance energy requirement at near-zero specific growth rates. Appl Environ Microbiol. 2016;82:4570–83. 27208115 10.1128/AEM.00638-16 PMC4984280

[bib60] Reifenberger E , Boles E, Ciriacy M. Kinetic characterization of individual hexose transporters of Saccharomyces cerevisiae and their relation to the triggering mechanisms of glucose repression. Eur J Biochem. 1997;245:324–33. 9151960 10.1111/j.1432-1033.1997.00324.x

[bib62] Rupp S , Summers E, Lo HJ et al. MAP kinase and cAMP filamentation signaling pathways converge on the unusually large promoter of the yeast *FLO11* gene. EMBO J. 1999;18:1257–69. 10064592 10.1093/emboj/18.5.1257 PMC1171216

[bib63] Schneider CA , Rasband WS, Eliceiri KW. NIH Image to ImageJ: 25 years of image analysis. Nat Methods. 2012;9:671–5. 22930834 10.1038/nmeth.2089 PMC5554542

[bib64] Schneper L , Düvel K, Broach JR. Sense and sensibility: nutritional response and signal integration in yeast. Curr Opin Microbiol. 2004;7:624–30. 15556035 10.1016/j.mib.2004.10.002

[bib65] Simon JM , Giresi PG, Davis IJ et al. Using formaldehyde-assisted isolation of regulatory elements (FAIRE) to isolate active regulatory DNA. Nat Protoc. 2012;7:256–67. 22262007 10.1038/nprot.2011.444 PMC3784247

[bib66] Smukalla S , Caldara M, Pochet N et al. *FLO1* is a variable green beard gene that drives biofilm-like cooperation in budding yeast. Cell. 2008;135:726–37. 19013280 10.1016/j.cell.2008.09.037 PMC2703716

[bib67] Soneson C , Love MI, Robinson MD. Differential analyses for RNA-seq: transcript-level estimates improve gene-level inferences. F1000Res. 2015;4:1521. 26925227 10.12688/f1000research.7563.1 PMC4712774

[bib68] Song Q , Kumar A. An overview of autophagy and yeast pseudohyphal growth: integration of signaling pathways during nitrogen stress. Cells. 2012;1:263–83. 24710476 10.3390/cells1030263 PMC3901118

[bib69] Stein LD , Mungall C, Shu S et al. The generic genome browser: a building block for a model organism system database. Genome Res. 2002;12:1599–610. 12368253 10.1101/gr.403602 PMC187535

[bib70] Sturmberger L , Chappell T, Geier M et al. Refined *Pichia pastoris* reference genome sequence. J Biotechnol. 2016;235:121–31. 27084056 10.1016/j.jbiotec.2016.04.023 PMC5089815

[bib71] Tatebayashi K , Tanaka K, Yang HY et al. Transmembrane mucins Hkr1 and Msb2 are putative osmosensors in the *SHO1* branch of yeast HOG pathway. EMBO J. 2007;26:3521–33. 17627274 10.1038/sj.emboj.7601796 PMC1949007

[bib72] Teunissen AW , Steensma HY. Review: the dominant flocculation genes of *Saccharomyces cerevisiae* constitute a new subtelomeric gene family. Yeast. 1995;11:1001–13. 7502576 10.1002/yea.320111102

[bib73] Vadaie N , Dionne H, Akajagbor DS et al. Cleavage of the signaling mucin Msb2 by the aspartyl protease Yps1 is required for MAPK activation in yeast. J Cell Biol. 2008;181:1073–81. 18591427 10.1083/jcb.200704079 PMC2442203

[bib74] Valli M , Tatto NE, Peymann A et al. Curation of the genome annotation of *Pichia pastoris* (Komagataella phaffii) CBS7435 from gene level to protein function. FEMS Yeast Res. 2016;16:fow051. 27388471 10.1093/femsyr/fow051

[bib75] Verstrepen KJ , Klis FM. Flocculation, adhesion and biofilm formation in yeasts. Mol Microbiol. 2006;60:5–15. 16556216 10.1111/j.1365-2958.2006.05072.x

[bib76] Westman JO , Nyman J, Manara RMA et al. A novel chimaeric flocculation protein enhances flocculation in *Saccharomyces cerevisiae*. Metab Eng Commun. 2018;6:49–55. 29896447 10.1016/j.meteno.2018.04.001 PMC5994806

[bib77] Wickham H , Hester J, Francois R. readr: Read Rectangular Text Data. 2015. https://www.springer.com/gp/book/9780387981413 (21 February 2018, date last accessed).

[bib78] Wright RM , Repine T, Repine JE. Reversible pseudohyphal growth in haploid *Saccharomyces cerevisiae* is an aerobic process. Curr Genet. 1993;23:388–91. 8319293 10.1007/BF00312623

[bib79] Zaman S , Lippman SI, Zhao X et al. How *Saccharomyces* responds to nutrients. Annu Rev Genet. 2008;42:27–81. 18303986 10.1146/annurev.genet.41.110306.130206

[bib80] Zhang Y , Liu T, Meyer CA et al. Model-based analysis of ChIP-Seq (MACS). Genome Biol. 2008;9:R137. 18798982 10.1186/gb-2008-9-9-r137 PMC2592715

