# Supplementary material for: Pseudohyphal differentiation in Komagataella phaffii: investigating the FLO gene family
Source: FEMS Yeast Res. 2020 Aug 7;20(5):foaa044. doi: 10.1093/femsyr/foaa044 (PMC7419694; doi:10.1093/femsyr/foaa044)
Supplement: foaa044_Supplemental_File [file foaa044_supplemental_file.pdf]

Supplementary File to

Pseudohyphal differentiation in *Komagataella phaffii*: investigating the *FLO* gene family

De Sonakshi, Rebnegger Corinna, Moser Josef, Tatto Nadine, Graf Alexandra B, Mattanovich Diethard, Gasser Brigitte

Supplementary tables

Table S1. Members of the *K. phaffii FLO* gene family

The following table lists the names of all annotated *FLO* genes and additional genes that show low similarity to flocculins, along with their feature names and identified conserved domains

Gene name	Feature	Conserved domains
FLO11	PP7435_Chr2-0267	FLO11 domain, Flocculin type 3 repeat
FLO5-1	PP7435_Chr3-1389	PA14/GLEYA domain, Flocculin type 3 repeat
FLO5-2	PP7435_Chr1-1228	PA14/GLEYA domain, Herpes BLLF1
FLO100	PP7435_Chr1-1587	PA14/GLEYA domain
FLO200	PP7435_Chr3-1226	PA14/GLEYA domain
FLO300	PP7435_Chr4-1020	PA14/GLEYA domain
FLO400	PP7435_Chr4-0865	Flocculin type 3 repeat
FLO8	PP7435_Chr4-0252	LiSH domain
BSC1	PP7435_Chr1-1549	FLO11 domain, Cellulose Binding Domain, Herpes BLLF1
PP7435_Chr1-2104	PP7435_Chr1-2104	Flocculin type 3 repeat
PP7435_Chr3-1237	PP7435_Chr3-1237	Flocculin type 3 repeat
PP7435_Chr4-0629	PP7435_Chr4-0629	Herpes BLLF1
PP7435_Chr4-1013	PP7435_Chr4-1013	PA14/GLEYA domain, Flocculin type 3 repeat
PP7435_Chr2-0004	PP7435_Chr2-0004	None identified

Table S2. RNA-Seq data for *FLO* genes when *K. phaffii* CBS7435 is grown in standard M2 medium supplemented with 5 g/L glucose (Ata Ö *et al.* 2018)

ID	Gene name	Wild type average tpm
**PP7435_Chr2-0267**	*FLO11*	1.1
**PP7435_Chr1-1389**	*FLO5-1*	300.6
**PP7435_Chr4-0865**	*FLO400*	25.7
**PP7435_Chr1-1549**	*BSC1*	9.1
**PP7435_Chr3-1228**	*FLO5-2*	118.3
**PP7435_Chr1-1587**	*FLO100*	0.0
**PP7435_Chr3-1226**	*FLO200*	58.0
**PP7435_Chr4-1020**	*FLO300*	176.5
**PP7435_Chr1-2104**	PP7435_Chr1-2104	568.9
**PP7435_Chr3-1237**	PP7435_Chr3-1237	224.7
**PP7435_Chr2-0004**	PP7435_Chr2-0004	17.7
**PP7435_Chr4-0629**	PP7435_Chr4-0629	337.6
**PP7435_Chr4-1013**	PP7435_Chr4-1013	395.1
**PP7435_Chr4-0252**	*FLO8*	3.1
**PP7435_Chr3-0993**	*ACT1*	182.5

Table S3. Genes that are significantly up- or downregulated (criteria adjusted p-value<0.05; log_2 fold-change below -1 or above 1) in the *K. phaffii* wild-type strain compared to the *flo8Δ* strain identified by RNA-Seq

CATEGORY	GENES
**Upregulated**	*PP7435_Chr1-0004, PHM7, IME2, HSP104, RIM4, MRN1, PP7435_Chr1-0172, PP7435_Chr1-0236, FAR1, PP7435_Chr1-0252, GPA1, PP7435_Chr1-0483, STE4, MIH1, SOR1, PRM1, ADH7, ADY2-4, HAC1, RME1, CHS7, PBP2, PP7435_Chr1-0819, FIG1, TPO3, PP7435_Chr1-1005, PP7435_Chr1-1074, URC1, PP7435_Chr1-1116, PP7435_Chr1-1117, PMP3-2, CWC21, PP7435_Chr1-1165, MNN14, PP7435_Chr1-1233, PP7435_Chr1-1387, PP7435_Chr1-1388,* ***FLO5-1****, PP7435_Chr1-1393, PpBMT3, SOA1 , PP7435_Chr1-1482, PP7435_Chr1-1496, GTT1, PP7435_Chr2-0218,* ***FLO11****, PDR15, PP7435_Chr2-0355, MF(ALPHA)1, MNN4-3, PP7435_Chr2-0638, FUS3, EXG1, PP7435_Chr2-0908, AOC1-1, GSC2, PP7435_Chr2-1044, PP7435_Chr2-1097, PTP2, MSC1, PP7435_Chr2-1246, PP7435_Chr2-1294, QUP4, PP7435_Chr2-2001, PP7435_Chr2-2705, DLD1, ALD6-2, YPS1-5, STE6-1, AGP2-2, SST2, PP7435_Chr3-0415, AQY1, PP7435_Chr3-0480, RAM1, TOS8, PP7435_Chr3-0762, PP7435_Chr3-0863, SSA3, PP7435_Chr3-1213, AYF1, SOA1, ALS3,* ***PP7435_Chr3-1237****, STE3, PP7435_Chr3-1710, FMP45, MATalpha1, PP7435_Chr4-0101, PP7435_Chr4-0336, HSP12, PP7435_Chr4-0344, KAR4, PP7435_Chr4-0631, PP7435_Chr4-0632, PP7435_Chr4-0690, PP7435_Chr4-0827,* ***FLO400****, STE2, PP7435_Chr4-0976, PP7435_Chr4-1001, PP7435_Chr4-1007, FET4-2, OPT1-1, MATa2, PP7435_Chr4-1576, STE12*
**Downregulated**	*PEX11C, PMP20, PMP3-1, TRS23, PP7435_Chr2-0423, PP7435_Chr2-0527, PP7435_Chr2-1495, COX9, PP7435_Chr2-1968, ATP15, PP7435_Chr2-2540, SIT1-1, FMO1-2, MUP1-2, PP7435_Chr3-1224, PP7435_Chr3-1545, AOX1, TMA10, PP7435_Chr4-1014, RKI1-2, NU4M*

Table S4. Enriched GO terms for genes showing differential regulation in the *flo8Δ* strain compared to the wild-type strain

GO Term	Genes
Multi-organism process	*PRM1, KAR4, SST2, FLO11, STE12, GPA1, FUS3, FIG1, STE3, STE2, FAR1, STE4, YPS1*
Multi-organism cellular process	*PRM1, KAR4, SST2, FLO11, STE12, GPA1, FUS3, FIG1, STE3, STE2, FAR1, STE4, YPS1*
Reproduction	*KAR4, SST2, RIM4, IME2, MSC1, FUS3, HAC1, FIG1, STE3, STE4, YPS1, PRM1, MIH1, ADY2, AQY1, MATalpha1, STE12, GPA1, RME1, FMP45, GSC2, STE2, FAR1*
Positive regulation of reproduction	*SST2, MIH1, GPA1, FUS3, STE3, FAR1, STE4, STE2, YPS1*
Response to pheromone	*KAR4, SST2, STE12, GPA1, FUS3, STE3, STE2, FAR1, STE4, STE6*
Unannotated or no paralog in *S. cerevisiae*	*PP7435_Chr4-0336, PMP20, PP7435_Chr1-1387, PP7435_Chr2-1294, PP7435_Chr2-0638, PP7435_Chr1-1388, PP7435_Chr1-0004, PP7435_Chr4-0631, PP7435_Chr1-0252, FLO400, PP7435_Chr3-1224, PpBMT3, PP7435_Chr1-1482, ALS3, PP7435_Chr2-2705, PP7435_Chr2-1097, PP7435_Chr2-0908, PP7435_Chr2-0527, PP7435_Chr2-0423, PP7435_Chr4-0632, PP7435_Chr1-1005, PP7435_Chr4-1007, PP7435_Chr4-0344, PP7435_Chr1-1393, PP7435_Chr2-2540, PP7435_Chr4-1001, PP7435_Chr4-1576, MATa2, PP7435_Chr1-1496, PP7435_Chr2-0355, URC1, PP7435_Chr1-0819, PP7435_Chr1-0236, PP7435_Chr1-1116, PP7435_Chr1-1233, PP7435_Chr2-0522, PP7435_Chr3-1237, PP7435_Chr4-0976, PP7435_Chr1-1117, PEX11C, AYF1, PP7435_Chr3-0480, AOC1, PP7435_Chr3-1545, NU4M, PP7435_Chr3-1710, PP7435_Chr1-0483, PP7435_Chr4-0101, PP7435_Chr1-0172, PP7435_Chr2-0218, AOX1, PP7435_Chr3-0415, QUP4, PP7435_Chr2-1246, PP7435_Chr2-2001, PP7435_Chr1-1165, PP7435_Chr1-1074, PP7435_Chr2-1968, PP7435_Chr4-1014, PP7435_Chr3-1213, PP7435_Chr4-0827, PP7435_Chr2-1495*

Table S5. Genes proximal to which FAIRE-Seq peaks that undergo stable changes upon switching to slow growth rate were identified (Category I & Category II)

CATEGORY	GENES
**CATEGORY I**	*PP7435_Chr1-0819, PBP2, PFK2, SSY5, **FLO5-1**, MNN4-3, LDB19, PMT2, PIB2-2, PP7435_Chr2-1074, MRP1, PEX3, LRP1, PP7435_Chr3-0094, PP7435_Chr3-1161, PP7435_Chr3-1162, PP7435_Chr3-1163, SKN7, SCH9, PSA1-1, PP7435_Chr3-0308, PP7435_Chr3-1688, FBP1, **FLO400**, HOM3, PP7435_Chr4-1884*
**CATEGORY II**	*KAP104, PP7435_Chr1-0014, PP7435_Chr1-0015, PP7435_Chr1-3402, RPP0, RGP1, RSN1, TEL1, RPL23A, PP7435_Chr1-0148,SNU71, MSB2, PP7435_Chr1-1895, PP7435_Chr1-0287,OSH2, NTE1, PP7435_Chr1-0302, ULP2, PP7435_Chr1-0366,TMA20, IST3, PP7435_Chr1-0365, UTP8, DCG1, RIF1, ADY2-1, RPL12B, MAK10, PP7435_Chr1-0510, AAT1, PP7435_Chr1-0522, PP7435_Chr1-0521, ATP7, PP7435_Chr1-3435, LCD1, RPL37A, PLM2, UBC6, MVD1, AIM29, MGM101, NUM1, PMT4, KTR4, PP7435_Chr1-0825, BRR2, CIR2, NRD1, RCI50, PP7435_Chr1-2599, PP7435_Chr1-0834, ASC1, PP7435_Chr1-2654, HFD1, CHO2, TYW3, GTR2, SPO11, NMA1, RPL34A, MMF1, RCF2, DCW1, DSC2, PAC10, RPS19B, RPL18B, PP7435_Chr1-1180, SNO1, SNZ3, AIM17, PP7435_Chr1-1224, MCA1, PP7435_Chr1-1223, FLR1, GPI1, FRE8,VPS35, RPS29A,TSR1, PP7435_Chr1-1394, PP7435_Chr1-1395, RPS2, NAB2, PP7435_Chr1-1517, RPL36A, MRPL24, GYP5, PP7435_Chr1-1534,TEF2, RAD53, PEP4, PP7435_Chr3-0074, VTI1, MET5, MPC3, TIM8, PP7435_Chr3-0083, ISC1, PP7435_Chr3-0084, GLT1, PWP1, RAO1, DAS2, PP7435_Chr3-0359, GPM1, NSP1, PP7435_Chr3-0376, PP7435_Chr3-0375, PP7435_Chr3-1732, PP7435_Chr3-0374, AQY1, RPS5, BUD2, SEC14, PP7435_Chr3-0704, PP7435_Chr3-0703, YMD8, STP2, PP7435_Chr3-0842, CCC1, PP7435_Chr3-2304, ERG6, VPS53, AIF1-3, PP7435_Chr3-0916, COQ2, RPS30A, PP7435_Chr3-0931, PP7435_Chr3-0932, SOA1_8, YCS4, PP7435_Chr3-0973, MIT1, SAP30, CDC5, PP7435_Chr3-0979, TOP1, RRP9, PP7435_Chr3-1023, RMD5, BIG1, PP7435_Chr3-1115, PP7435_Chr3-1117, TFC6, PP7435_Chr3-1212, PP7435_Chr2-0058, SEC53, RPL10, BUD20, AYR1, GDT1, SUN4, GPC1, PUF4, EFT1, ULP1, EBP2, PP7435_Chr2-1462, INO1, PER1, CBF5, PST1, SAM50-1, PP7435_Chr2-0215, PP7435_Chr2-0218, PEX13, YDC1, PP7435_Chr2-0266, PP7435_Chr2-0293, SOA1_5, NGG1, SDH4, PP7435_Chr2-0296, PP7435_Chr2-0310, CYC3, PDA1, NUP2, RPS25A, PP7435_Chr2-0368, OCA1, OST2, PP7435_Chr2-0366, RSR1, JIP5, RIM9, PP7435_Chr2-0418, MEF1, PUT2, RPS18A, YHP1, PSP1, YML6, ADA2, RPS17B, EGD2, DNF2, LNP1, PP7435_Chr2-0525, STR2, ATP18, DAL2, PP7435_Chr2-0595, VMA11, PP7435_Chr2-0594, ALP100, GAR1, PP7435_Chr2-0634, SAK1, PP7435_Chr2-0642, GFA1, YCG1, DIP2, DCN1, PP7435_Chr2-0680, APC2, GRX6, RPS11B, HAP1, ZTA1, PP7435_Chr2-0965, ASE1, PP7435_Chr2-0985, PP7435_Chr2-0986, ECM4, PP7435_Chr2-0987, MET6, NIK100, GOS1, PKC1, EFT2, UBA2, IDH2, BMH2, ARX1, PP7435_Chr2-1270, YET3, SEC8, NIT1, JLP1-2, PP7435_Chr2-2738, PP7435_Chr2-1290, SLA2-2, SUI1-1, CWC25-1, PP7435_Chr4-0066, CIN5-2, SLA2-1, CWC25-2, SUI1-2, GID7, CUE2, GAL10, PDR12, UTP22, FMS1-2, AOX1, PP7435_Chr4-0143, NOP1, PP7435_Chr4-1224, NCP1, CPS1, CAR1-2, PRO2, ISR1, PP7435_Chr4-0320, GPI11, PP7435_Chr4-0318, UBX3, ADE12, MSS2, LGD1,*

	PP7435_Chr4-0397, YCK2, PHO86, PP7435_Chr4-0403, SPO75, CRH1, TUB2, VBA1-2, KSS1, PP7435_Chr4-0444, ATM1, MSC2, PP7435_Chr4-0660, MIG1-1, PP7435_Chr4-0680, ARO10-2, NUXM, ABZ2, TRX1, CAX4, PP7435_Chr4-0736, PP7435_Chr4-2159, CEX1, ERG2, RNR2, RPL8A, RIO2, DCP2, RPL8A, DED1, NAM8, CPA1, RPL3, YPQ2, RLM1, KIP2, RPL8A, ERG4, THI11, PP7435_Chr4-0953, RPL8A, LDH1, PP7435_Chr4-1983, HEM14, VID24, UTH1, PP7435_Chr4-0973, PP7435_Chr4-0974, RPL22A, PP7435_Chr4-0976, CYC1, SSY1, PP7435_Chr4-1002, PP7435_Chr4-1001, PP7435_Chr4-1003

Table S6. List of Primers

The table below lists all the primers used in this study

Primers for Golden Gate Assembly of knock-out cassettes. The restriction sites for Golden Gate Cloning are underlined.

flo11Δ	5' homologous region FS AB	GATAGGTCTCCGATCGTCAGGAGATTGATGAGATT<br>GATAGGTCTCCCCGGGACTGGTGTAGAAGCAAAA
	3' homologous region FS CD	GATAGGTCTCCAATTCAGTTGCACTTCTCTTCA<br>GATAGGTCTCCAGCTTCCGTTTAAATCACACCC
flo5-1Δ	5' homologous region FS AB	GATAGGTCTCCGATCGCTTGAGAAGAAACGGTAGTTTCACAATG<br>GATAGGTCTCCCCGGGAAACTTCACGCCTCAACGCAATGCG
	3' homologous region FS CD	GATAGGTCTCCAATTAGTCATCAGGCTCTGACTGGGCAG<br>GATAGGTCTCCAGCTCGTATGCAACGGAGCACACGGAACAGTG
flo400Δ	5' homologous region FS 12	GATAGAAGACTCGGAGGTAGCGGAATGTGTGAAACTCCAC<br>GATAGAAGACTCCATGGGACTAATTGGCTGTTTATATACCGTG
	3' homologous region FS 34	GATAGAAGACTCGCTTCTCATTGTAGCCACATTGCACAGC<br>GATAGAAGACTCAGCGGAGAGGGCAGGAGCAAAAAACTTCATCACATTGAA[illegible]
Marker genes	FS BC	GATAGGTCTCCCCGGGTACGCTGCAGGTCGACAAC<br>GATAGGTCTCCAATTAGTGGATCTGATATCACCTA
	FS 23	GATAGAAGACTCCATGGTACGCTGCAGGTCGACAAC<br>GATAGAAGACTCAAGCAGTGGATCTGATATCACCTA

Primers for amplification of split marker cassettes and verification of positive knock-outs

flo11Δ	FLO11 _KO_ampl_fwd	**GTCAGGAGATTGATGAGATT**
	NatMX_rev	TGCGTTGACGTTGGTGAC
	NatMX_fwd	GTCCTTCACCACCGACACC
	FLO11_KO_ampl_rev	TCCGTTTAAATCACACCC
	FLO11_KO_ctr_fwd	GTAGGTCGTTCACATTTTGTGGCT

	FLO11_KO_ctr_rev	ATTGGGACCTGTTAGGGTGACA
flo5-1Δ	FLO5-1_KO_ampl_fwd	GCATCCAAATTGCTTGAGAAGAAACG
	KanMX_rev	CAGGAACACTGCCAGCGCATCAAC
	KanMX_fwd	GATGTTACAGATGAGATGGTCAGAC
	FLO5-1_KO_ampl_rev	GAACCGAGTTCATGTAGCAGATGTTAC
	FLO5-1_KO_ctr_fwd	GTGATGACAATTCAATACAGGACCCGGT
	FLO5-1_KO_ctr_rev	GAAAGTTGTTGGAAGGTCATTGCTC
flo400Δ	FLO400_KO_ampl_fwd	GTAGCGGAATGTGTGAAACTCCAC
	KanMX_rev	CAGGAACACTGCCAGCGCATCAAC
	KanMX_fwd	GATGTTACAGATGAGATGGTCAGAC
	FLO400_KO_ampl_rev	GAGAGGGCAGGAGCAAAAAACTTCATCACATTGAAG
	FLO400_KO_ctr_fwd	CAGAATAATAGCGTATAAGGGCTTTCAACACCACG
	FLO400_KO_ctr_rev	GTGGATGGCTTCTCAGTGGTTGGTGGAGGA

Primers for assembling and amplifying donor fragments for Flo5-1-egfp and *Flo400-egfp* reporter strains

Flo5-1-egfp	5' homologous region FS AB	GGTCTCCGATCGCTCTTCGACGATGCATGCAATTTCGCAATAG<br>GGTCTCTTCACAGCAGCAGCTTGTCCATGTACACCAAGGACAC
	Egfp FS BC	GGTCTCCGTGAGCAAGGGCGAGGAGCTGTTC<br>GGTCTCTTTTCAGCAGCAGCCTTGTACAGCTCGTCCATGCCGAGAG
	3' homologous region FS CD	GGTCTCTGAAAGTGGTAACGGTACCACTAGTG<br>GGTCTCTAATTGCTCTTCCGTAGTGGCATCTAGGCTTGTTCCAG
	Donor amplification	ATGCATGCAATTTCGCAATAGTTCGTTGC<br>GTGGCATCTAGGCTTGTTCCAGCGAGCAC
	Confirmation of integration	GTAGTTTCACAATGTGGCAGTTTGATTAC<br>GAAGGTCATTGCTCGCCCAGTAAG
Flo400-egfp	5' homologous region FS AB	GAAGACCTGGAGGAATGCGGATGGGGTGCGATTGATTTTCG<br>GAAGACCTTCACAGCAGCAGCATCAGCAAAGGCTG
	Egfp FS BC	GAAGACTCGTGAGCAAGGGCGAGGAGCTGTTC<br>GAAGACCTCAGAAGCAGCAGCCTTGTACAGCTCGTCCATGCCGAG

	3' homologous region Fragment 1 FS C	GAAGACCTTCTGGAGAGTTCACTCTTTGGAAC<br>GAAGACCTTGTTGTCTCGTCGTCCTCCTCTGG
	3' homologous region Fragment 2 FS D	GAAGACTCAACAACAACCGATGATCCAACCG<br>GAAGACCTAGCGGAATGCGAATACAGTGTTAATAATAAGC
	Donor amplification	GGCAAAGTCTTGAGGAGGTGCAAAAACGG<br>AGTAGCACCAGTAATTTTTATGTCGGAAGGAGGGAAGGTTG
	Confirmation of integration	GTCGTTCACATTTTGTGGCTATAATCG<br>GGTCTCTAATTGAAGACTACCCAAG

qRT PCR primers

FLO11	AGTCCACACACCATTGACAACTGC<br>ACACCAGTAACTATTGTAGCAACTGAGC
FLO5-1	CAGGCAAGTGATGACAATTCAATACAGGAC<br>CGTTTGGTATCCCATAAAGTGTTGTGGTG
FLO5-2	GCTATTTCAAAGCTGCTGTTTCAGGAGAC<br>AGCGAATAGTCTGTTGGTGCTTGATCC
FLO400	GGAGCTGAAGGTTCGGTAGAAT<br>GTTCTGCACCCTCACAAATGT
FLO100	TCACGACCGGTGGTACGACTA<br>ATTTGGCACAACGTGGCTAGGT
FLO200	GTCTGGGACAAGTGACAACA<br>ATGGTTGCATTCACGTACGA
BSC1	TGAGGAATCCACAGAGGAATCCACAT<br>TGGATTCCTCAGCATCCTCAGTAGA
ACT1	CCTGAGGCTTTGTTCCACCCATCT<br>GGAACATAGTAGTACCACCGGACATAACGA

Supplementary figures

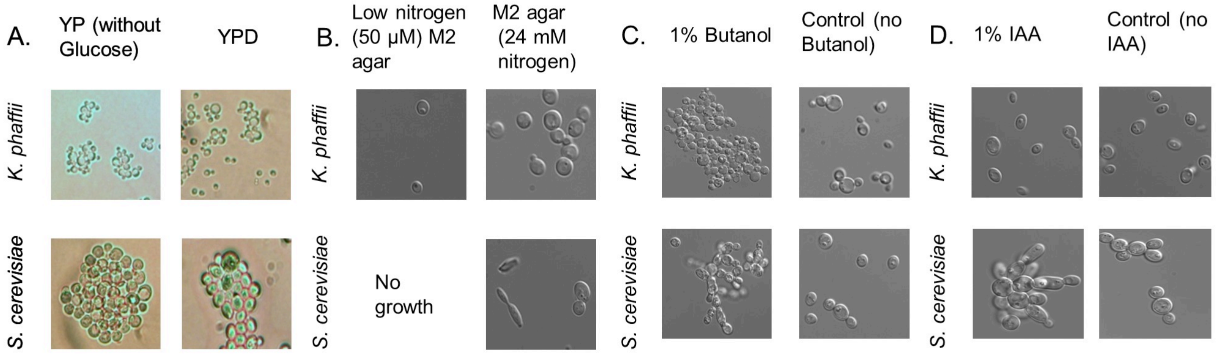

Fig. S1. *K. phaffii* does not switch to pseudohyphal phenotype when cultivated under conditions that are able to trigger pseudohyphal differentiation in *S. cerevisiae*. The above figures show microscope pictures of *K. phaffii* and *S. cerevisiae* after cultivation under different conditions. (A) YP agar (without glucose) and YPD (with glucose) (B) Standard and low nitrogen containing minimal agar (C) In presence of 1% butanol D. In presence of 1% isoamyl alcohol

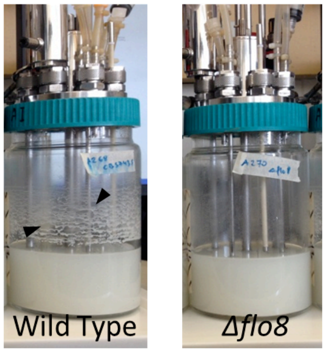

Fig. S2. Surface growth on bioreactors observed in the wild-type samples at slow growth rates. Surface growth was observed on the walls of the bioreactor when the dilution rate was switched to 0.05 h^{-1} for the wild-type strain. In case of the *Δflo8* strain this phenotype was not observed suggesting that the transcription factor Flo8 controls this phenotype.

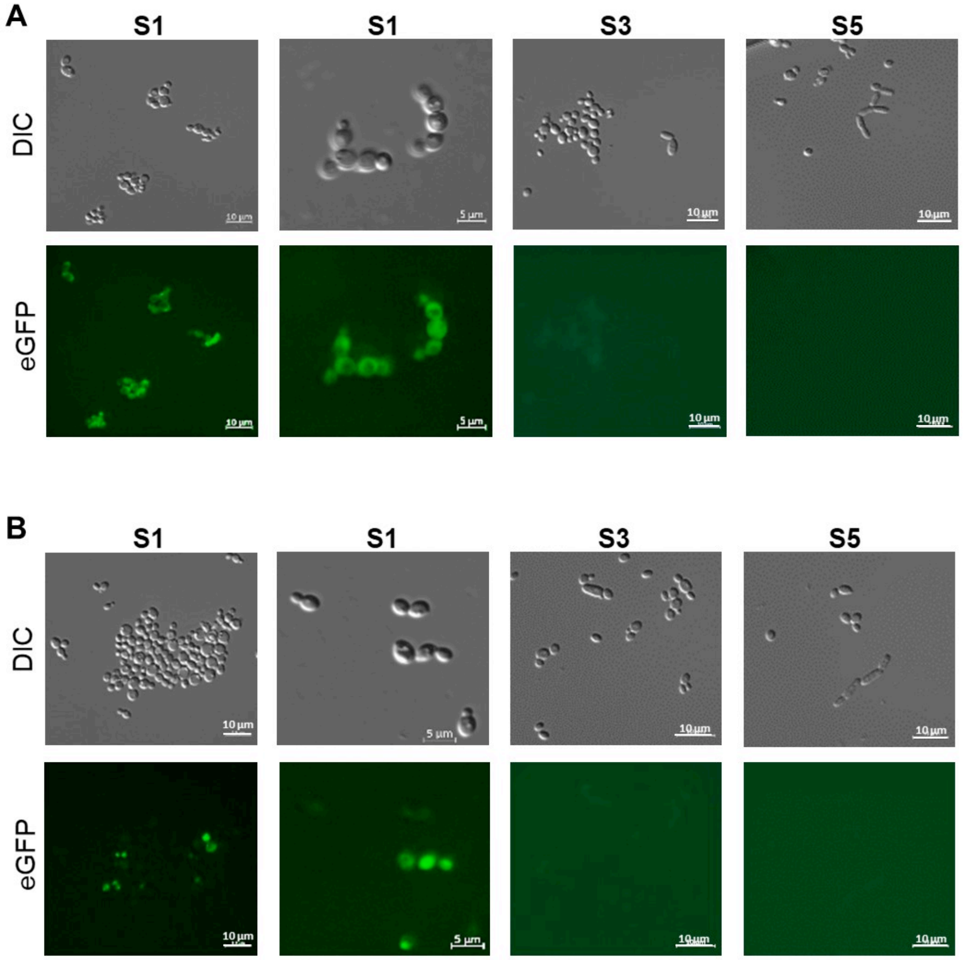

Fig. S3. Microscope pictures showing the expression of Flo5-1 and Flo400 by Flo5-1:eGFP and Flo400-eGFP expressing reporter strains. The upper panel shows the DIC image and the lower panel shows the corresponding eGFP image.

Visualization of (A) Flo400:eGFP and (B) Flo5-1:eGFP at the three different sampling points S1, S3 and S5, and a zoom at S1 for better visualization. Absence of fluorescence at S3 and S5 in both (A) and (B) indicates that the proteins are not expressed at these sampling time points

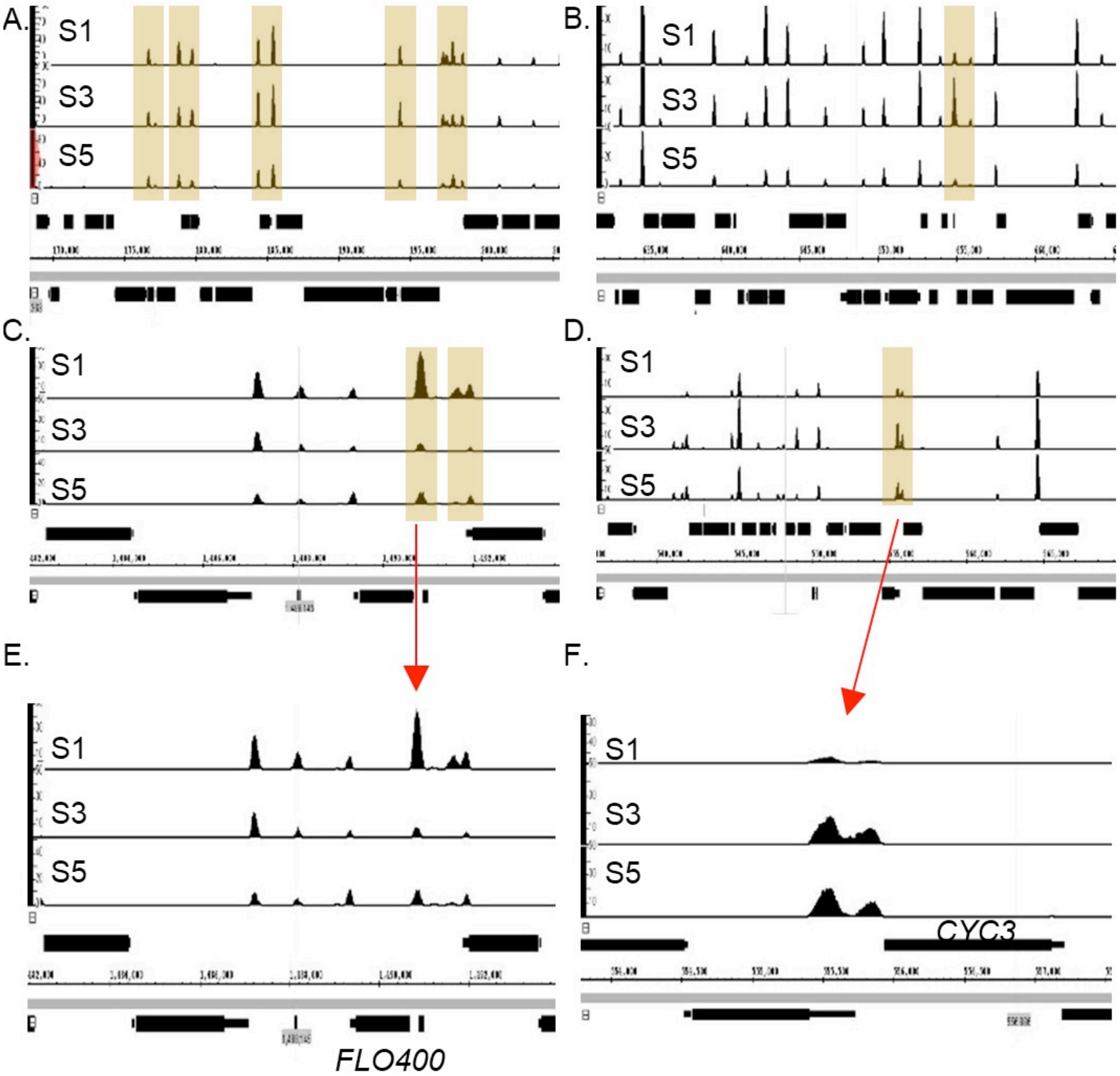

Fig. S4. Screenshots of Integrated Genome Browser (IGB) showing various chromosome regions representing different types of peaks encountered in the FAIRE-Seq data. S1, S3 and S5 are the three sampling time-points. (A) Peaks that do not change over the different stages of chemostat; (B) Peaks that are not detected in S1 and S5 but only detected in S3; (C,E) Peaks that are detected at S1, but not at S3 and S5 (corresponding to chromatin regions that are stably closed after switching to the slow μ = Category I, e.g. *FLO400*); and (D,F) Peaks that are not detected at S1, but appear in S3 and S5 (corresponding to stably opened chromatin regions after switching to the slow μ = Category II, e.g. *CYC3*).
